# Supplementary material for: Characterizing College Science Assessments: The Three-Dimensional Learning Assessment Protocol
Source: PLoS One. 2016 Sep 8;11(9):e0162333. doi: 10.1371/journal.pone.0162333 (PMC5015839; doi:10.1371/journal.pone.0162333)
Supplement: S1 Protocol — (DOCX) [file pone.0162333.s002.docx]

**Characterizing College Science Assessments: The Three-Dimensional Learning Assessment Protocol**

James T. Laverty, Sonia M. Underwood, Rebecca L. Matz, Lynmarie A. Posey, Justin H. Carmel, Marcos D. Caballero, Cori L. Fata-Hartley, Diane Ebert-May, Sarah E. Jardeleza, Melanie M. Cooper

**Supporting Information: 3D-LAP**

# Purpose

The Three-Dimensional Learning Assessment Protocol (3D-LAP) is a protocol designed for two purposes:

1. To characterize assessment tasks and entire assessments as having the potential to elicit evidence that students have engaged with the three dimensions identified in *A Framework for K-12 Science Education: Practices, Crosscutting Concepts, and Core Ideas*.
2. To help instructors develop or modify existing assessment tasks so that they have the potential to elicit evidence of students engaging with the three dimensions.

To accomplish these goals, the 3D-LAP describes criteria that can be used to determine if an assessment task has the potential to elicit evidence of students engaging with the three dimensions. Because the 3D-LAP was developed primarily for college-level assessments, it was necessary to modify some aspects of the three dimensions outlined in the Framework. In particular, we modified a few of the scientific practices and developed new sets of core ideas appropriate for college-level biology, chemistry, and physics courses.

##

## Characterizing Assessment Tasks and Assessments

The 3D-LAP criteria are applied to assessment tasks as they are written. That is, the 3D-LAP in no way attempts to determine how a student would respond to an assessment task but instead focuses on what students are explicitly asked to do. To characterize an assessment task, the user should identify if the task meets all of the criteria for each of the scientific practices, agrees with any of the descriptions for the crosscutting concepts, or aligns with any of the ideas listed in the core ideas

Assessments (e.g., exams) are characterized by first characterizing each individual task on the assessment. Figure 1 shows a possible representation for the coding of tasks on an assessment. This can be used to characterize the extent to which students are being assessed on each of the three dimensions.

When coding assessments with the 3D-LAP, it is important to take into account clusters of questions that are related to each other as parts of a larger task. For the purposes of coding, we define a cluster to be a set of tasks that are explicitly linked by a diagram, context, question stem, or similar construct. Both individual tasks belonging to a cluster and the clusters themselves may be coded. This distinction is important because coding the cluster as a whole may meet the criteria for some dimensions while none of the individual parts satisfy those criteria when considered separately. For example, tasks 9-12 in Figure 1 belong to a cluster, which is coded as being three-dimensional, even though none of the individual tasks is coded with a crosscutting concept. Examples of coding tasks, including clusters, can be found in the Supporting Information: Exemplars document.

| Question Number | 1 | 2 | 3 | 4 | 5 | 6 | 7 | 8 | 9 | 10 | 11 | 12 | 9-12 | 13 | 14 | 15 |
| --- | --- | --- | --- | --- | --- | --- | --- | --- | --- | --- | --- | --- | --- | --- | --- | --- |
| Scientific Practices |  |  |  |  |  |  |  |  |  |  |  |  |  |  |  |  |
| Crosscutting Concepts |  |  |  |  |  |  |  |  |  |  |  |  |  |  |  |  |
| Core Ideas |  |  |  |  |  |  |  |  |  |  |  |  |  |  |  |  |
| Figure 1: Data obtained by applying the 3D-LAP to individual tasks in an assessment. The top row identifies each task (one of which is a cluster) on the assessment, while the bottom three rows indicate whether that task does (colored with blue, green, or red) or does not (white) have the potential to elicit evidence that a student has engaged with that dimension. In this example, questions 1-12 are *selected response tasks* and questions 13-15 are *constructed response tasks*. | | | | | | | | | | | | | | | | |

###

## Constructing or Revising Assessment Tasks

There are a number of ways to design assessment tasks that have the potential to elicit evidence that a student has engaged with three-dimensional learning. For example, authors might choose a scientific practice, crosscutting concept, and core idea that they wish to assess, using the criteria in the 3D-LAP to guide their assessment task writing. Alternatively, authors might examine existing questions and think about how to modify a task (or make it part of a cluster) so it has the potential to elicit evidence of three-dimensional learning.

# Scientific Practices

Development of the criteria for identifying the scientific practices in assessments was guided by the question “What specific components must be present in a task in order for it to potentially elicit evidence of student engagement with a given practice?” The results are sets of criteria that specify the information the task must provide and the actions that the task prompts students to perform as part of their answer (note, this is not necessarily the same as the actions that students actually perform).

There are significant distinctions in the nature of the evidence provided regarding students’ use of scientific practices in constructed response versus selected response assessment tasks. Ideally, engaging in scientific practices requires that a student construct their responses, thus providing *a priori* evidence of their thinking. Conversely, selected response tasks must provide the student with a set of possible answers. It is impossible to get direct evidence that a student has engaged with ***Constructing*** *Explanations* (for example) when they are **selecting** an answer choice.

However, because many introductory science courses have large enrollments, instructors often choose to use selected response tasks on assessments. Selected response tasks require fewer resources to score and allow for quicker turnaround of feedback to students. For these reasons, we acknowledge that selected response assessment tasks are often necessary for large courses. Therefore, in addition to the *Scientific Practices Criteria for Constructed Response Assessment Tasks*, we also developed *Scientific Practices Criteria for Selected Response Assessment Tasks.* Questions that meet the latter sets of criteria provide the best evidence that students are engaging with the scientific practices without requiring students to construct their own responses.

# Crosscutting Concepts and Core Ideas

The criteria developed for the Crosscutting Concepts and Core Ideas differ in their nature from those presented for the Scientific Practices. Crosscutting Concepts and Core Ideas are constructs, in contrast with the Scientific Practices, which describe actions associated with doing science. As such, descriptions of how students are asked to engage with each Crosscutting Concept and Core Idea capture their essence better than lists of specific actions that a student must take in responding to an assessment task.

This document contains three sets of Core Ideas for introductory college courses, one each for biology, chemistry, and physics. These core ideas differ from the ones in the Framework because a) they represent college level (not K-12) content and the organization of content by discipline, and b) they were developed in consultation with the disciplinary faculty at Michigan State University.

For each discipline, we list Core Ideas and provide descriptions to clarify what each of the negotiated core ideas encompasses and to provide assistance in coding. The core ideas are characterized by guidelines that experts within a discipline can use. We argue that these core ideas have a relatively well-negotiated meaning within their disciplines, however, it may well be that others would prefer a different set of core ideas. Indeed, we encourage faculty to initiate such conversations at their own institutions.

## Scientific Practices Criteria for Constructed Response Assessment Tasks

### Asking Questions

Student is asked to generate a scientific question about a real world event, observation, phenomenon, data, scenario, or model.

1. Question gives an event, observation, phenomenon, data, scenario, or model.
2. Question asks student to generate an empirically testable question about the given event, observation, phenomenon, data, scenario, or model.

### Developing and Using Models

Student is given or asked to construct a mathematical, graphical, computational, symbolic, or pictorial representation and use it to explain or predict an event, observation, or phenomenon.

1. Question gives an event, observation, or phenomenon for the student to explain or make a prediction about.
2. Question gives a representation or asks student to construct a representation.
3. Question asks student to explain or make a prediction about the event, observation, or phenomenon.
4. Question asks student to provide the reasoning that links the representation to their explanation or prediction.

### Planning Investigations

Student is asked to design an experimental method or identify a set of observations that can be used to answer a scientific question or test a claim or hypothesis.

1. Question poses a scientific question, claim, or hypothesis to be investigated.
2. Question asks student to describe or design an investigation, or identify the observations required to answer the question or test the claim or hypothesis.
3. Question asks student to justify how their description, design, or observations can be used to answer the question or test the claim or hypothesis.

### Analyzing and Interpreting Data

Student is given a question, claim, or hypothesis and data collected from an experiment or observation and is asked to analyze the resulting data and interpret their meaning.

1. Question gives a scientific question, claim, or hypothesis to be investigated.
2. Question gives a representation of the data (e.g., table or graph, or list of observations) provided to answer the question or test the claim or hypothesis.
3. Question gives an analysis of the data or asks student to analyze the data.
4. Question asks student to interpret the results or assess the validity of the conclusions in the context of the scientific question, claim, or hypothesis.

### Using Mathematics and Computational Thinking

Student is asked to use mathematical reasoning or a calculation and interpret the results within the context of the given event, observation, or phenomenon.

1. Question gives an event, observation, or phenomenon.
2. Question asks student to perform a calculation or statistical test, generate a mathematical representation, or demonstrate a relationship between parameters.
3. Question asks student to give a consequence or an interpretation (not a restatement) in words, diagrams, symbols, or graphs of their results in the context of the given event, observation, or phenomenon.

### Constructing Explanations and Engaging in Argument from Evidence

Student is asked to provide reasoning based on evidence to support a claim.

1. Question gives an event, observation, or phenomenon.
2. Question gives or asks student to make a claim based on the given event, observation, or phenomenon.
3. Question asks student to provide scientific principles or evidence in the form of data or observations to support the claim.
4. Question asks student to provide reasoning about why the scientific principles or evidence support the claim.

### Evaluating Information

Student is asked to make sense of information or ideas presented to them.

1. Question gives an excerpt from a conversation, article, student solution, or video (or similar form of communication) that makes one or more assertions.
2. Question gives a conclusion about the validity of the assertion(s) made or asks student to make a conclusion about the validity of the assertion(s) or reconcile multiple assertions with each other.
3. Question asks student to provide reasoning to support their conclusion(s) about the validity of the assertion(s) or reconciliation with data, observations, or scientific principles.

##

##

## Scientific Practices Criteria for Selected Response Assessment Tasks

### Developing and Using Models

Student is given or asked to select a mathematical, graphical, computational, symbolic, or pictorial representation and select an appropriate explanation or prediction about an event, observation, or phenomenon based on the representation.

1. Question gives an event, observation, or phenomenon for the student to explain or make a prediction about.
2. Question gives a representation or asks student to select a representation.
3. Question asks student to select an explanation for or prediction about the event, observation, or phenomenon.
4. Question asks student to select the reasoning that links the representation to their explanation or prediction.

### Planning Investigations

Student is asked to select an appropriate design of an experimental method or an observation that can be used to answer a scientific question or test a claim or hypothesis.

1. Question poses a scientific question, claim, or a hypothesis to be investigated.
2. Question asks student to select a description of or a design for an investigation or select the observations that could be used to answer the question or test the hypothesis.
3. Question asks student to select a justification of how the description, design, or observations can be used to answer the question or test the claim or hypothesis.

### Analyzing and Interpreting Data

Student is given a question, claim, or hypothesis and data collected from an experiment or observation and is asked to select an interpretation of its meaning.

1. Question gives a scientific question, claim, or a hypothesis to be investigated.
2. Question gives a representation of data (table, graph, list of observations, etc.) provided to answer the question or test the claim or hypothesis.
3. Question asks student to select an interpretation of the results or an assessment of the validity of the conclusions in the context of the scientific question, claim, or hypothesis.

### Using Mathematics and Computational Thinking

Student is expected to perform a mathematical manipulation and asked to select an interpretation of the results within the context of a given event, observation, or phenomenon.

1. Question gives an event, observation, or phenomenon.
2. Question asks student to perform a calculation or statistical test, use a mathematical representation, or derive a relationship between parameters in order to obtain the correct answer.
3. Question asks student to select a consequence or an interpretation (not a restatement) in words, diagrams, symbols, or graphs of their results in the context of the given event, observation, or phenomenon.

### Constructing Explanations and Engaging in Argument from Evidence

Student is asked to select reasoning and evidence to support a claim.

1. Question gives an event, observation, or phenomenon.
2. Question gives or asks student to select a claim based on the given event, observation, or phenomenon.
3. Question asks student to select scientific principles or evidence in the form of data or observations to support the claim.
4. Question asks student to select the reasoning about why the scientific principles or evidence support the claim.

### Evaluating Information

Student is asked to make sense of information or ideas presented to them.

1. Question gives an excerpt from a conversation, article, student solution, or video (or similar form of communication) that makes one or more assertions.
2. Question gives a conclusion about the validity of the assertion(s) or asks student to select a conclusion about the validity of the assertion(s) or reconciliation of multiple assertions.
3. Question asks student to select reasoning to support their conclusion(s) about the validity of the assertion(s) or reconciliation with data, observations, or scientific principles.

# Crosscutting Concepts

## Patterns

To code an assessment task with Patterns, the question asks the student to identify patterns or trends emerging from three or more events, observations, or data.

##

## Cause and Effect: Mechanism and Explanation

To code an assessment task with Cause and Effect: Mechanism and Explanation, the question provides at most two of the following: 1) a cause, 2) an effect, and 3) the mechanism that links the cause and effect, and the student is asked to provide the other(s).

##

## Scale, Proportion, and Quantity

### Scale

To code an assessment task with Scale, the question asks the student 1) to compare objects, processes, or properties across size, time, or energy scales, or to dimensions of familiar objects, timescales, or energies or 2) to identify non-negligible/relevant interactions at various scales.

### Proportion and Quantity

To code an assessment task with Proportion and Quantity, the question asks the student to predict the response of one variable to changes in another or identify the relationship between two or more variables from data.

##

## Systems and System Models

To code an assessment task with Systems and System Models, the question asks the student to identify a system (by defining its components or boundaries), any assumptions made, and the surroundings (if necessary), and how the system and surroundings interact with each other.

##

## Energy and Matter: Flows, Cycles, and Conservation

To code an assessment task with Energy and Matter: Flows, Cycles, and Conservation, the question asks the student to describe the transfer or transformation of energy or matter within or across systems, or between a system and its surroundings, with explicit recognition that energy and/or matter are conserved.

##

## Structure and Function

To code an assessment task with Structure and Function, the question asks the student to predict or explain a function or property based on a structure, or to describe what structure could lead to a given function or property.

##

## Stability and Change

To code an assessment task with Stability and Change, the question asks the student to determine 1) if a system is stable and provide the evidence for this, or 2) what forces, rates, or processes make a system stable (static, dynamic, or steady state), or 3) under what conditions a system remains stable, or 4) under what conditions a system is destabilized and the resulting state.

# Core Ideas

## Biology Core Ideas

1. **Chemical and Physical Basis of Life**: Life processes are the result of regulated chemical and physical interactions and reactions governed by the laws of physics.
2. **Matter and Energy**: Free energy and matter are used in regulated processes that establish order, support growth and development, and control dynamic homeostasis.
   - Thermodynamically favorable reactions are coupled with thermodynamically unfavorable reactions in these regulated molecular processes.
   - Molecules and atoms from the environment are rearranged to build new molecules in these regulated molecular processes.
   - Photosynthesis, cellular respiration, and trophic dynamics are key processes mediating the cycling of matter and flow of energy at multiple scales (from cells to ecosystems).
3. **Cellular Basis of Life**: Cells are the fundamental units of all living things.
   - Cells are created from other cells.
   - Cells interact with other cells.
4. **Systems**: Ecosystems, organisms, tissues, and cells act as systems.
   - Dynamic molecular interactions result in the emergence of increasingly complex biological properties.
   - Changes in chemical and physical signals affect the structure and performance of cell systems.
   - Organisms interact with their abiotic and biotic environments at multiple scales for the purpose of obtaining resources. These interactions mediate movements of matter, energy, and information in ecological systems and are subject to physical and chemical laws.
     - The distribution and abundance of organisms -- as well as the direction, magnitude, and frequencies of their interactions -- can change in space and time and are linked to availabilities of matter, energy, and other resources.
   - Evolutionary histories, random/stochastic effects, and humans impact ecosystem structure, function, and dynamics.

1. **Structure and Function**: The functions and properties of ecosystems, organisms, tissues, cells, and biological molecules are determined by their structures.
   - At the molecular level, biology is based on dynamic, three-dimensional chemical and physical interactions.
2. **Information Flow, Exchange, and Storage**: Hereditary information is stored, used, and replicated.
   - DNA is the source of heritable information in a cell.
   - The growth and behavior of organisms is determined by the information contained in their genes and by the regulated expression of those genes.
     - The molecular structure and function of cells is regulated by gene expression.
     - Gene expression and protein activity are regulated by intracellular and extracellular signaling molecules that vary over time and depend on environmental conditions.
   - The transmission of DNA between generations follows predictable patterns.
     - Meiosis produces gametes for sexual reproduction.
     - Mendelian genetics predicts many patterns of inheritance.
     - Additional inheritance patterns are related to the assortment of linked genes, epigenetic changes, and the vertical and horizontal transfer of genetic information.
3. **Evolution:** Evolution drives the diversity and unity of life.
   - Evolution is the change in allele and/or genotypic frequencies in a population and is manifested in the changes in inherited characteristics over generations.
   - Evolutionary change is driven by natural selection and genetic drift. Variation, caused by random mutation and passed on by reproduction, provides the raw material for evolution.
   - Organisms are linked by lines of descent from common ancestry. Phylogenetic trees are hypotheses of the pattern of descent from a common ancestor which can be explored and tested.
   - Evolution affects the genetic makeup of populations over time and can lead to adaptation, speciation, and extinction.
   - Evolution can be tested using the Hardy-Weinberg Equilibrium as the null hypothesis.

## Chemistry Core Ideas

1. **Electrostatic and Bonding Interactions**: Attractive and repulsive electrostatic forces govern noncovalent and bonding (covalent and ionic) interactions between atoms and molecules. The strength of these forces depends on the magnitude of the charges involved and the distances between them.
   - Attractive noncovalent interactions (intermolecular forces) between atoms and molecules arise from interactions between transient, induced, and permanent dipoles.
   - Atoms also interact through electrostatic forces to form chemical bonds, which have greater stability (lower energy) than the separated atoms. Nonpolar covalent bonding and ionic bonding represent the limits in a continuum of bonding interactions with polar covalent bonds falling in-between. Electrons in the highest energy orbitals with the largest spatial extent (valence electrons) are used to form bonds.
   - Covalent and ionic bonding interactions are typically stronger than noncovalent (intermolecular) interactions.
2. **Atomic/Molecular Structure and Properties**: The macroscopic physical and chemical properties of a substance are determined by the three-dimensional structure, the distribution of electron density, and the nature and extent of the noncovalent interactions between particles.
   - The three-dimensional structure of a molecule is determined by the atoms that make up the molecule, the repulsions between the bonding and nonbonding electrons, and the distribution of the valence electrons within the molecule.
   - The properties of a molecule are determined by the interactions and associated energy and entropy changes that occur when molecules interact.
3. **Energy**: Energy changes are either the cause or the consequence of change in chemical systems, which can be considered on different scales and can be accounted for by conservation of the total energy of the system of interest and the surroundings.
   - Macroscopic: Changes in phase and reactions of collections of atoms and/or molecules are accompanied by energy changes that result from energy changes on the atomic/molecular scale.
     - Typically, these energy changes are observed by a change in temperature or a phase change.
     - Temperature is determined by the average kinetic energy of collections of atoms and/or molecules.
   - Atomic/Molecular: Kinetic and potential energy changes occur when atoms and molecules interact. Energy is released to the surroundings when bonds or attractive noncovalent interactions form, and conversely energy is required to break bonds or noncovalent interactions.
     - The overall energy change that accompanies a chemical reaction is the net result of the energy added to break bonds and the energy released when new bonds form.
     - Energetic barriers along the path between reactants and products (reaction coordinate) determine the rates of reaction.
   - Quantum Mechanical Energy Levels and Changes: Energy levels are quantized in atoms and molecules resulting in discrete energies for transitions between energy levels. This is a direct consequence of the wave-particle duality of electrons and other subatomic particles.
     - Energy levels are quantized in atoms and molecules.
     - Evidence for the quantization of energy levels comes from spectroscopy. The features of observed spectra correspond to transitions between quantized energy states and directly provide the energy difference between initial and final energy states.
     - The electromagnetic radiation that interacts with atoms and molecules in spectroscopic measurements also exhibits wave-particle duality.
4. **Change and Stability in Chemical Systems**: Energy and entropy changes, the rates of competing processes, and the balance between opposing forces govern the fate of chemical systems.
   - Change: Change in chemical systems results from the natural evolution of the system or occurs in response to a perturbation to the system.
   - Stability: Stability in a chemical system results from the balancing of forces or the rates of competing processes. While we often ascribe a move towards “stability” as the driving force for a particular change in a system, these underlying ideas are the mechanism by which stability is achieved.
   - Gibbs Energy and Entropy: The thermodynamic function Gibbs free energy captures the roles of enthalpy and entropy in determining the direction of change of a chemical system. The Gibbs free energy is a proxy for the total entropy change of the universe (system + surrounding) based on properties of the system.

## Physics Core Ideas

1. **Interactions Can Cause Changes in Motion**: Changes in an object’s motion are the result of interactions between it and one or more other objects. Multiple interactions between an object and its surroundings can result in a predictable change in motion.
   - All macroscopic forces are the result of gravitational or electromagnetic interactions between particles.
   - Non-zero net forces cause changes in linear momentum.
   - Non-zero net torques cause changes in angular momentum.
   - Changes in linear and angular momentum can be used to predict the motion of objects.
2. **Energy is Conserved**: Energy comes in many forms and can be transformed from one form to another within a given system or transferred between systems.
   - ΔE = W + Q; the total energy of a system changes based on a system’s interactions with its surroundings. These interactions can be separated into the work done on/by the surroundings (W) and the thermal energy exchanged with the surroundings (Q).
   - Work is due to macroscopic interactions between objects.
   - Thermal energy exchanged with the surroundings is due to molecular level interactions between constituent particles.
3. **Exchanges of Energy increase Total Entropy**: Multiparticle systems tend toward states that are more statistically likely to occur. At a macroscopic scale, this can be described by concepts such as entropy, temperature, and pressure.
   - Energy is quantized.
   - Energy can be distributed within a system of particles in many different ways (microstates).
   - Left alone, systems tend towards macrostates with a larger number of associated microstates.
   - The total entropy of the universe is always increasing.
   - The total energy change of a system at a given temperature is related to the mechanical work done (pressure-volume work) on/by the system and the change in entropy of that system at the given temperature.
4. **Interactions are Mediated by Fields**: Fields are generated by charges/masses. Fields affect charges/masses. In circuits, fields induce currents.
   - All charges generate electric fields.
   - Moving charges generate magnetic fields.
   - Net electric fields affect all charges.
   - Net magnetic fields affect moving charges.
   - Masses generate gravitational fields.
   - Net gravitational fields affect all masses.
5. **Energy, Momentum, Angular Momentum, and Information can be Transported without a Net Transfer of Matter**: Mechanical waves move through matter. Electromagnetic waves can move through vacuum or matter. Properties of waves can be used to parameterize the information or amount of energy, momentum, or angular momentum is transported.
   - Mechanical waves cause matter to oscillate in order to transfer energy, momentum, angular momentum, and information without a net transfer of material.
   - Electromagnetic radiation exhibits both wave and particle properties, and these models can be used to investigate or explain different phenomena.
