# Supplementary material for: Characterizing College Science Assessments: The Three-Dimensional Learning Assessment Protocol
Source: PLoS One. 2016 Sep 8;11(9):e0162333. doi: 10.1371/journal.pone.0162333 (PMC5015839; doi:10.1371/journal.pone.0162333)
Supplement: S1 Supporting Information — (DOCX) [file pone.0162333.s003.docx]

**Characterizing College Science Assessments: The Three-Dimensional Learning Assessment Protocol**

James T. Laverty, Sonia M. Underwood, Rebecca L. Matz, Lynmarie A. Posey, Justin H. Carmel, Marcos D. Caballero, Cori L. Fata-Hartley, Diane Ebert-May, Sarah E. Jardeleza, Melanie M. Cooper

**Supporting Information: Exemplars**

This document contains assessment tasks from each discipline and how they are coded using the 3D-LAP, as agreed upon by the authors. Some questions are not three-dimensional, but all contain a scientific practice. Every discipline-specific section contains at least one example of a constructed response task and a selected response task for each scientific practice. While assessment tasks can have the potential to elicit evidence of students engaging with multiple scientific practices, crosscutting concepts, or core ideas, we only list one for each exemplar for the sake of simplicity. Table 1 summarizes the question type and identifies the scientific practice, crosscutting concept, and core idea with which each exemplar is coded. For selected response tasks an asterisk (*) indicates the correct answer. Each discipline-specific section has the following format:

*Question number*

*Question stem and answer choices (where applicable)*

| CRITERIA | RATIONALE |
| --- | --- |
| Scientific Practice: *Name of Scientific Practice* | |
| 1. *Criteria for the Scientific Practice* | 1. *The components of the exemplar that meet the criteria for the Scientific Practice* |
| Crosscutting Concept: *Name of Crosscutting Concept* | |
| *Criteria for the Crosscutting Concept* | *The components of the exemplar that meet the criteria for the Crosscutting Concept* |
| Core Idea: *Name of Core Idea* | |
| *Criteria for Core Idea* | *The components of the exemplar that meet the criteria for the Core Idea* |

| Table 1. This table summarizes the scientific practices, crosscutting concepts, and core ideas that were coded for each exemplar using the 3D-LAP. “CR” indicates constructed response tasks, and “SR” indicates selected response tasks. “NA” (not applicable) indicates that the exemplar does not have the potential to elicit evidence of students engaging with that particular dimension. | | | | |
| --- | --- | --- | --- | --- |
| Task Number | Task Type | Scientific Practice | Crosscutting Concept | Core Idea |
| Biology | | | | |
| B1 | CR | Asking Questions | Systems and System Models | Systems |
| B2 | CR | Developing and Using Models | Structure and Function | Structure and Function |
| B3 | CR | Planning Investigations | NA | Chemical and Physical Basis of Life |
| B4 | CR | Analyzing and Interpreting Data | NA | Matter and Energy |
| B5 | CR | Using Mathematics and Computational Thinking | Proportion and Quantity | Evolution Drives the Diversity and Unity of Life |
| B6 | CR | Constructing Explanations and Engaging in Argument from Evidence | Structure and Function | Information Flow, Exchange, and Storage |
| B7 | CR | Evaluating Information | NA | Information Flow, Exchange, and Storage |
| B8 | SR | Developing and Using Models | Cause and Effect: Mechanism and Explanation | Structure and Function |
| B9 | SR | Planning Investigations | NA | Evolution Drives the Diversity and Unity of Life |
| B10 | SR | Analyzing and Interpreting Data | Patterns | NA |
| B11 | SR | Using Mathematics and Computational Thinking | Scale | Chemical and Physical Basis of Life |
| B12 | SR | Constructing Explanations and Engaging in Argument from Evidence | Cause and Effect: Mechanism and Explanation | NA |
| B13 | SR | Evaluating Information | NA | Matter and Energy |
| Chemistry | | | | |
| C1 | CR | Asking Questions | NA | NA |
| C2 | CR | Developing and Using Models | Cause and Effect: Mechanism and Explanation | Atomic/Molecular Structure and Properties |
| C3 | CR | Planning Investigations | Energy and Matter: Flows, Cycles, and Conservation | NA |
| C4 | CR | Analyzing and Interpreting Data | Patterns | Energy - Quantum Mechanical Energy Levels and Changes |
| C5 | CR | Using Mathematics and Computational Thinking | Energy and Matter: Flows, Cycles, and Conservation | NA |
| C6 | CR | Constructing Explanations and Engaging in Argument from Evidence | Cause and Effect: Mechanism and Explanation | Energy - Quantum Mechanical Energy Levels and Changes |
| C7 | CR | Evaluating Information | Systems and System Models | Electrostatic and Bonding Interactions |
| C8 | SR | Developing and Using Models | Cause and Effect: Mechanism and Explanation | Energy - Atomic/Molecular |
| C9 | SR | Planning Investigations | Proportion and Quantity | NA |
| C10 | SR | Analyzing and Interpreting Data | Energy and Matter: Flows, Cycles, and Conservation | Energy - Macroscopic |
| C11 | SR | Using Mathematics and Computational Thinking | Scale | NA |
| C12 | SR | Constructing Explanations and Engaging in Argument from Evidence | Cause and Effect: Mechanism and Explanation | Electrostatic and Bonding Interactions |
| C13 | SR | Evaluating Information | Structure and Function | Atomic/Molecular Structure and Properties |
| Physics | | | | |
| P1 | CR | Asking Questions | NA | NA |
| P2 | CR | Developing and Using Models | Systems and System Models | Interactions Can Cause Changes in Motion |
| P3 | CR | Planning Investigations | NA | Interactions are Mediated by Fields |
| P4 | CR | Analyzing and Interpreting Data | Patterns | NA |
| P5 | CR | Using Mathematics and Computational Thinking | Proportion and Quantity | Interactions are Mediated by Fields |
| P6 | CR | Constructing Explanations and Engaging in Argument from Evidence | Structure and Function | Interactions Can Cause Changes in Motion |
| P7 | CR | Evaluating Information | Cause and Effect: Mechanism and Explanation | Interactions Can Cause Changes in Motion |
| P8 | SR | Developing and Using Models | Systems and System Models | Interactions Can Cause Changes in Motion |
| P9 | SR | Planning Investigations | NA | Interactions are Mediated by Fields |
| P10 | SR | Analyzing and Interpreting Data | Patterns | Interactions are Mediated by Fields |
| P11 | SR | Using Mathematics and Computational Thinking | Proportion and Quantity | Interactions are Mediated by Fields |
| P12 | SR | Constructing Explanations and Engaging in Argument from Evidence | Structure and Function | Interactions are Mediated by Fields |
| P13 | SR | Evaluating Information | NA | Interactions Can Cause Changes in Motion |

##

##

## Biology Exemplars

B1

**Biology Example 1:** As you observed in class, termites respond to various stimuli such as scent. For example, they will follow lines drawn on paper from certain pens. Develop a testable research question that stems from your observations of termite behavior in response to ink scent. Identify one assumption that you are making in your question.

| CRITERIA | RATIONALE |
| --- | --- |
| Scientific Practice: Asking Questions | |
| 1. Question gives an event, observation, phenomenon, data, scenario, or model. 2. Question asks student to generate an empirically testable question about the given event, observation, phenomenon, data, scenario, or model. | 1. Question stem states “observations of termite behavior.” 2. Question asks student to “Develop a testable research question”. |
| Crosscutting Concept: Systems and System Models | |
| To code an assessment task with Systems and System Models, the question asks the student to identify a system (by defining its components or boundaries), any assumptions made, and the surroundings (if necessary), and how the system and surroundings interact with each other. | The question asks the student to develop a question about how termites interact with different kinds of ink and to identify an assumption implicit in the question (e.g., that different termites will behave consistently or that different types of paper do not affect termite behavior). |
| Core Idea: Systems | |
| Organisms interact with their abiotic and biotic environments at multiple scales (molecules to ecosystems) for the purpose of obtaining resources. | The question asks the student to develop a question about how termites (an organism) interacts with ink, an abiotic component of the system |

B2

**Biology Example 2:** Create a diagram that shows the molecular structure of the lipid bilayer in a typical cell membrane. Use the diagram to explain why oxygen (O_2_) can easily pass through the membrane but sodium ions (Na^+^) cannot.

| CRITERIA | RATIONALE |
| --- | --- |
| Scientific Practice: Developing and Using Models | |
| 1. Question gives an event, observation, or phenomenon for the student to explain or make a prediction about. 2. Question gives a representation or asks student to construct a representation. 3. Question asks student to explain or make a prediction about the event, observation, or phenomenon. 4. Question asks student to provide the reasoning that links the representation to their explanation or prediction. | 1. Question gives a phenomenon: “oxygen (O_2_) can easily pass through the membrane but sodium ions (Na^+^) cannot”. 2. Question asks student to “create a diagram that depicts the molecular structure of the lipid bilayer in a typical cell membrane”. 3. Question asks student to “explain why”. 4. Question asks student to “use the diagram to explain”. |
| Crosscutting Concept: Structure and Function | |
| To code an assessment task with Structure and Function, the question asks student to predict or explain a function or property based on a structure, or to describe what structure could lead to a given function or property. | The question asks student to explain the differences between how oxygen and sodium ions pass through a cell membrane using the molecular structure of a lipid bilayer. |
| Core Idea: Structure and Function | |
| The functions and properties of ecosystems, organisms, tissues, cells, and biological molecules are determined by their structures. | The question asks the student to explain the differences between how oxygen and sodium ions pass through a cell membrane using the molecular structure of a lipid bilayer. |

B3

**Biology Example 3:** You are planning an experiment to test the hypothesis that growing raspberry bushes in slightly acidic versus neutral soil will increase fruit production. You have 17 bushes at your disposal. Describe the experimental design, including the rationale for the experimental design. Explain why it is important to consider the pH of the soil.

| CRITERIA | RATIONALE |
| --- | --- |
| Scientific Practice: Planning Investigations | |
| 1. Question poses a scientific question, claim, or hypothesis to be investigated. 2. Question asks student to describe or design an investigation, or identify the observations required to answer the question, to answer the question or test the claim or hypothesis. 3. Question asks student to justify how their description, design, or observations can be used to answer the question or test the claim or hypothesis. | 1. Question poses a hypothesis: “growing raspberry bushes in slightly acidic versus neutral soil will increase fruit production.” 2. Question asks student to “Describe the experimental design”. 3. Question asks student to provide “the rationale for the experimental design”. |
| Crosscutting Concept: Not Applicable | |
| Core Idea: Chemical and Physical Basis of Life | |
| Life processes are the result of regulated chemical and physical interactions and reactions governed by the laws of physics. | The question asks the student to consider how the acidity or neutrality of soil could impact plant growth. |

B4

**Biology Example 4:** In the Rocky Mountains, water availability to plants varies significantly. For example, slopes that face south are much drier than north-facing exposures, and steep slopes are drier than gentle slopes. We also know that plants with low solute potential can grow in drier environments. Examine the two figures below. Note that ninebark is *Physocarpus* (genus in the woody plant family Rosaceae). Based on the data represented, if Sitka alder and ninebark were competing for water, which plant would gain water more effectively and why?


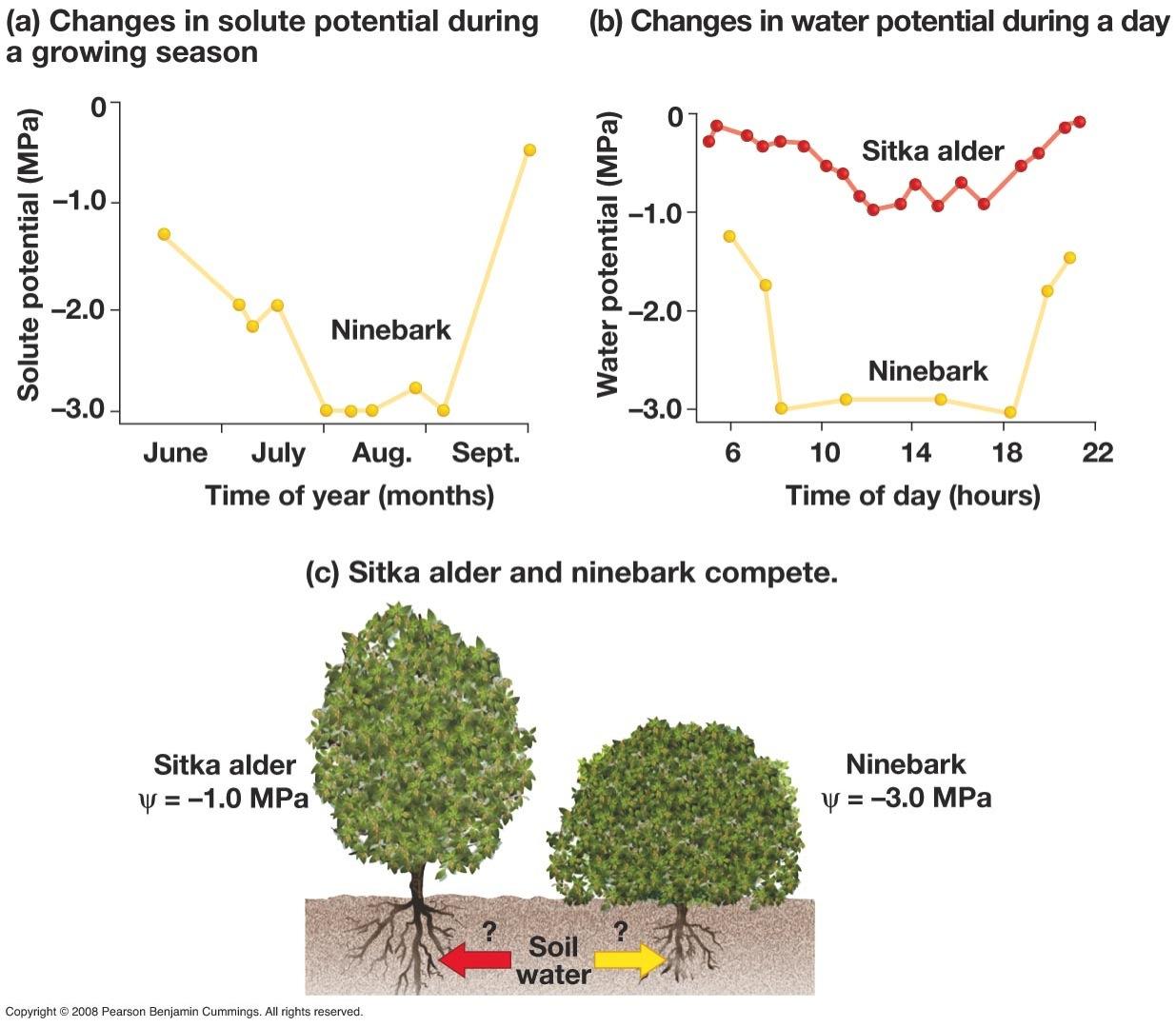


Freeman, Scott, *Biological Science*, 3rd Edition, © 2008. Reprinted by permission of Pearson Education, Inc., New York, New York.

| CRITERIA | RATIONALE |
| --- | --- |
| Scientific Practice: Analyzing and Interpreting Data | |
| 1. Question gives a scientific question, claim, or hypothesis to be investigated. 2. Question gives a representation of the data (e.g., table or graph, or list of observations) provided to answer the question or test the claim or hypothesis. 3. Question gives an analysis of the data or asks student to analyze the data. 4. Question asks student to interpret the results or assess the validity of the conclusions in the context of the scientific question, claim, or hypothesis. | 1. Question gives the question “which plant would gain water more effectively and why?” 2. Question provides two graphs of data on solute potential and water potential. 3. Question asks student to determine which plant would gain water more effectively. 4. Question asks student to rationalize why that plant would gain water more effectively. |
| Crosscutting Concept: Not Applicable | |
| Core Idea: Matter and Energy | |
| Free energy and matter are utilized in regulated molecular processes that establish order, support growth and development, and regulate dynamic homeostasis in cells. | The question asks the student to use data to determine which plant species would compete for water and therefore grow more effectively. |

B5

*Credited to Andrew M. Jarosz.*

**Biology Example 5:** Ninety percent of a wolf’s diet on Isle Royale is moose. Moose on Isle Royale have a mean weight of 480 kilograms (kg), but weight is a highly variable trait (variance = 10,201 kg^2^) (see figure).


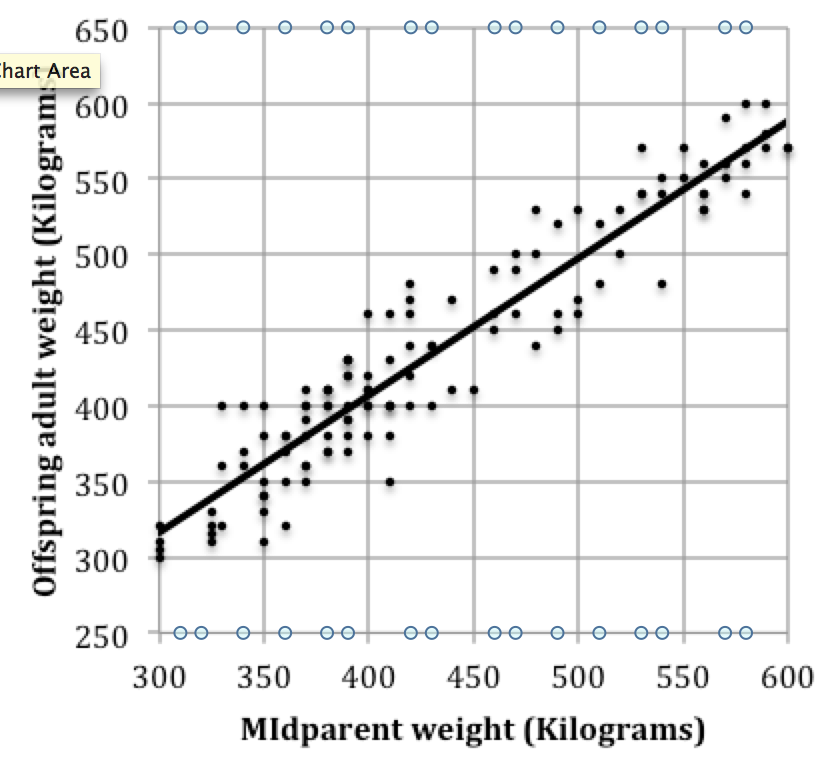


1. Calculate the heritability for moose weight on Isle Royale.
2. A Canadian moose population, which resides on the mainland directly across from Isle Royale, has a calculated heritability for body weight of 0.65. Predict which of these two populations (Isle Royale or Canadian) will evolve faster if wolves are causing similar selective pressure for increased body weight at both sites. Assume that the selection differential is 0.017. Justify your prediction using evidence.

| CRITERIA | RATIONALE |
| --- | --- |
| Scientific Practice: Using Mathematics and Computational Thinking | |
| 1. Question gives an event, observation, or phenomenon. 2. Question asks student to perform a calculation or statistical test, generate a mathematical representation, or demonstrate a relationship between parameters. 3. Question asks student to give a consequence or an interpretation (not a restatement) in words, diagrams, symbols, or graphs of their results in the context of the given event, observation, or phenomenon. | 1. Question notes the phenomenon of moose evolution. 2. Question asks student to “Calculate the heritability for moose weight on Isle Royale.” 3. Question asks student to “Predict which of these two populations (Isle Royale or Canadian) will evolve faster” and “Justify your prediction using evidence.” |
| Crosscutting Concept: Proportion and Quantity | |
| To code an assessment task with Proportion and Quantity, the question asks the student to predict the response of one variable to changes in another or identify the relationship between two or more variables from data. | The question asks the student to predict which herd of moose will evolve more quickly based on calculated heritability. |
| Core Idea: Evolution Drives the Diversity and Unity of Life | |
| Evolution is the change in allele and/or genotypic frequencies in a population and is manifested in the changes in inherited characteristics over generations. | The question asks the student to use information about how heritability of a trait, moose weight, will affect the evolution of future generations. |

B6

**Biology Example 6:** Consider the claim that DNA replication occurs with high fidelity. Use the double-stranded structure of DNA as evidence to explain how the structure provides a mechanism for high-fidelity replication.

| CRITERIA | RATIONALE |
| --- | --- |
| Scientific Practice: Constructing Explanations and Engaging in Argument from Evidence | |
| 1. Question gives an event, observation, or phenomenon. 2. Question gives or asks student to make a claim based on the given event, observation, or phenomenon. 3. Question asks student to provide scientific principles or evidence in the form of data or observations to support the claim. 4. Question asks student to provide reasoning about why the scientific principles or evidence support the claim. | 1. Question gives the phenomenon of DNA replication. 2. Question gives the “claim that DNA replication occurs with high fidelity”. 3. Question asks student to “Use the double-stranded structure of DNA as evidence”. 4. Question asks student to “explain how the structure provides a mechanism for high-fidelity replication”. |
| Crosscutting Concept: Structure and Function | |
| To code an assessment task with Structure and Function, the question asks the student to predict or explain a function or property based on a structure, or to describe what structure could lead to a given function or property. | The question asks the student to use the double-stranded structure of DNA to help explain why DNA can replicate with high fidelity. |
| Core Idea: Information Flow, Exchange, and Storage | |
| DNA is the source of heritable information in a cell. | The question asks the student to explain the mechanism by which DNA can act as the source of heritable information in cells. |

B7

**Biology Example 7:** This image was acquired by conducting a Google Images search on the term “chromosome”. Is this model an accurate or inaccurate representation of the genetic information contained in a chromosome? Defend your answer by providing reasons (evidence) that support your claim. Draw and label a representation that you think is better.

| 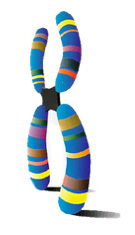 |
| --- |
| Image: http://reflexions.ulg.ac.be/cms/c_7338/en/chromosome |

| CRITERIA | RATIONALE |
| --- | --- |
| Scientific Practice: Evaluating Information | |
| 1. Question gives an excerpt from a conversation, article, student solution, or video (or similar form of communication) that makes one or more assertions. 2. Question gives a conclusion about the validity of the assertion(s) made or asks student to make a conclusion about the validity of the assertion(s) or reconcile multiple assertions with each other. 3. Question asks student to provide reasoning to support their conclusion(s) about the validity of the assertion(s) or reconciliation with data, observations, or scientific principles. | 1. Question provides the chromosome image from an internet search. 2. Question asks student to determine if this image is an accurate model of a chromosome. 3. Question asks student to provide reasons why the model is or is not accurate, and to draw a new representation that is more accurate. |
| Crosscutting Concept: Not Applicable | |
| Core Idea: Information Flow, Exchange, and Storage | |
| DNA is the source of heritable information in a cell. | The question asks the student to interpret why the information represented on the chromosome image is not appropriate given that the function of replicated DNA is to provide identical copies to daughter cells. |

B8

*Credited to Jon R. Stoltzfus.*
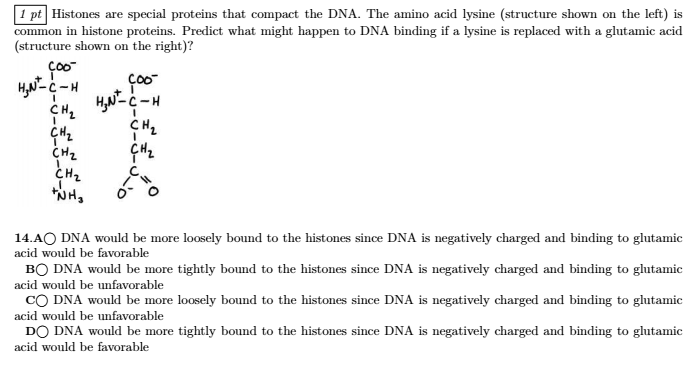


**Biology Example 8:** Histones are special proteins that compact DNA. The amino acid lysine (structure on the left) is common in histone proteins. Predict and explain what might happen to DNA binding if most lysines in a histone were replaced with glutamic acids (structure on the right)?

1. DNA would be more loosely bound to the histones since the negatively charged DNA backbone would NOT interact with glutamic acid residues. *
2. DNA would be more loosely bound to the histones since the positively charged DNA backbone would NOT interact with glutamic acid residues.
3. DNA would be more tightly bound to the histones since the negatively charged DNA backbone would interact with glutamic acid residues.
4. DNA would be more tightly bound to the histones since the positively charged DNA backbone would interact with the glutamic acid residues.

| CRITERIA | RATIONALE |
| --- | --- |
| Scientific Practice: Developing and Using Models | |
| 1. Question gives an event, observation, or phenomenon for the student to explain or make a prediction about. 2. Question gives student a representation or asks student to select a representation. 3. Question asks student to select an explanation for or prediction about the event, observation, or phenomenon. 4. Question asks student to select the reasoning that links the representation to their explanation or prediction. | 1. Question gives the observation that “Histones are special proteins that compact the DNA” 2. Question gives the structures of two amino acids. 3. Question asks student select an answer to “Predict … what might happen to DNA binding if most lysines in a histone were replaced with glutamic acids”. 4. Question asks student to select an answer to “explain what might happen to DNA binding if a lysine is replaced with a glutamic acid”. |
| Crosscutting Concept: Cause and Effect: Mechanism and Explanation | |
| To code an assessment task with Cause and Effect: Mechanism and Explanation, the question provides at most two of the following: 1) a cause, 2) an effect, and 3) the mechanism that links the cause and effect, and the student is asked to provide the other(s). | The question gives the cause (replacing lysine residues with glutamic acid residues) and asks for both the effect and mechanism. |
| Core Idea: Structure and Function | |
| The functions and properties of ecosystems, organisms, tissues, cells, and biological molecules are determined by their structures. | The question asks the student to use the amino acid structures to determine how changing one for the other in histones would impact the function of binding to DNA. |

B9

*Modified from original task written by Elena Bray Speth.*

**Biology Example 9:** A researcher asks the following question: Is Strain 1 bacteria or Strain 2 bacteria resistant to antibiotic X?

1. Which of the following experimental design options would be needed to test the question? *Choose all that apply*.
   1. A petri dish, with antibiotic X, with 10mL of Strain 1 added *
   2. A petri dish, without antibiotic X, with 10mL of Strain 1 added *
   3. A petri dish, with antibiotic X, with 10mL of Strain 2 added *
   4. A petri dish, without antibiotic X, with 10mL of Strain 2 added *
2. Why does the experimental design chosen reliably address the question? *Choose all that apply*.
   1. It tests if Strain 1 is resistant to antibiotic X. *
   2. It tests if Strain 2 is resistant to antibiotic X. *
   3. It tests if Strain 1 would grow without antibiotic present. *
   4. It tests if Strain 2 would grow without antibiotic present. *
3. Several days later you check the petri dish(es) with the Strain of bacteria that was not resistant to antibiotic X. You notice there are several colonies of the Strain of bacteria growing on the media in the petri dish(es). What best explains why the Strain is growing?
   1. Some of the Strain cells’ genes had mutations that allowed them to be resistant to antibiotic X, and over time the bacteria survived and divided into many more new cells. *
   2. Antibiotic X caused a mutation in the Strain cells’ genes that caused them to be resistant to antibiotic X, and over time the bacteria survived and divided into many more new cells.
   3. The original Strain cells are no longer present on the petri dish, but another strain of bacteria survived and divided into many more new cells.
   4. The antibiotic is no longer present on the petri dish, but the Strain cells survived and divided into many more new cells.

| CRITERIA | RATIONALE |
| --- | --- |
| Scientific Practice: Planning Investigations | |
| 1. Question poses a scientific question, claim, or a hypothesis to be investigated. 2. Question asks student to select a description of or a design for an investigation or select the observations that could be used to answer the question or test the hypothesis. 3. Question asks student to select a justification of how the description, design, or observations can be used to answer the question or test the hypothesis. | 1. Question stem asks if Strain 1 or 2 bacteria are resistant to antibiotic X. 2. Part 1 of the question asks student to select the samples needed to test the question. 3. Part 2 of the question asks student to select why the experimental design they developed adequately addresses the question. |
| Crosscutting Concept: Not Applicable | |
| Core Idea: Evolution Drives the Diversity and Unity of Life | |
| Evolutionary change is driven by natural selection and genetic drift. Variation, caused by random mutation and passed on by reproduction, provides the raw material for evolution. | Part 3 of the question asks the student how it is possible that resistant bacteria could be growing in a dish where only non-resistant bacteria were plated. |

B10

**Biology Example 10:** An experiment is performed to determine how a drug interferes with the cell cycle. All the cells are in G1 at the beginning of the experiment (timepoint 0). Samples from drug-treated and control cells are collected at 6 different time points, and the amount of DNA in each sample is determined. The results are shown in the table.

| Timepoint | Amount of DNA in control cells (pg/nucleus) | Amount of DNA in treated cells (pg/nucleus) |
| --- | --- | --- |
| 0 (G1) | 6.0 | 6.1 |
| 1 | 8.6 | 8.5 |
| 2 | 12.1 | 11.9 |
| 3 | 12.1 | 11.8 |
| 4 | 12.0 | 11.9 |
| 5 | 5.9 | 11.9 |
| 6 | 5.9 | 11.9 |

Based on the experimental results, which of the following provides the best explanation for how the drug interferes with the cell cycle?

1. The drug interferes at the G2/M checkpoint because the treated cells are not going through mitosis. *
2. The drug interferes at the G2/M checkpoint because the treated cells are repairing DNA damaged during synthesis.
3. The drug interferes at the G1/S checkpoint because the treated cells are not going through mitosis.
4. The drug interferes at the G1/S checkpoint because the treated cells are repairing DNA damaged during synthesis.

| CRITERIA | RATIONALE |
| --- | --- |
| Scientific Practice: Analyzing and Interpreting Data | |
| 1. Question gives a scientific question, claim, or a hypothesis to be investigated. 2. Question gives a representation of data (table, graph, list of observations, etc.) provided to answer the question or test the claim or hypothesis. 3. Question asks student to select an interpretation of the results or an assessment of the validity of the conclusions in the context of the scientific question, claim, or hypothesis. | 1. Question asks student for “the best explanation for how the drug interferes with the cell cycle?” 2. Question gives a table of data. 3. Question asks student to select the stage at which the cell cycle is being inhibited and why. |
| Crosscutting Concept: Patterns | |
| To code an assessment task with Patterns, the question asks the student to identify patterns or trends emerging from three or more events, observations, or data. | The question asks the student to identify a pattern in the experimental results that explains how the drug might interfere with the cell cycle. |
| Core Idea: Not Applicable | |

B11

**Biology Example 11:** Cells use 100,000 molecules of glucose per cubic micrometer (μm^3^) per second. A membrane protein called the glucose transporter moves glucose from outside the cell into the cytoplasm. Consider the relative number of glucose transporters for the two different cell types described in the table below.

| Cell Type | Cell Diameter |
| --- | --- |
| X | 20 μm |
| Y | 200 μm |

Which cell type would have a higher percentage of the cell membrane covered by glucose transporter molecules?

1. Cell type Y because as a cell gets larger, its volume increases at a faster rate than its surface area. *
2. Cell type Y because as a cell gets larger, the surface area increases leading to an increased percentage of glucose transporters.
3. Cell type X because as a cell gets larger, its volume increases at a faster rate than its surface area
4. Cell type X because as a cell gets larger, the surface area increases leading to an increased percentage of glucose transporters.

| CRITERIA | RATIONALE |
| --- | --- |
| Scientific Practice: Using Mathematics and Computational Thinking | |
| 1. Question gives an event, observation, or phenomenon. 2. Question asks student to perform a calculation or statistical test, use a mathematical representation, or derive a relationship between parameters in order to obtain the correct answer. 3. Question asks student to select a consequence or an interpretation (not a restatement) in words, diagrams, symbols, or graphs of their results in the context of the given event, observation, or phenomenon. | 1. Question describes a phenomenon: “Cells use 100,000 molecules of glucose per cubic micrometer (μm^3^) per second.” 2. Question requires student to relate surface area to volume. 3. Correct answer includes “its volume increases at a faster rate than its surface area.” |
| Crosscutting Concept: Scale | |
| To code an assessment task with Scale, the question asks the student 1) to compare objects, processes, or properties across size, time, or energy scales, or to dimensions of familiar objects, timescales, or energies or 2) to identify non-negligible/relevant interactions at various scales. | The question asks the student to compare a property (the relative number of glucose transport membrane proteins) for cells that differ substantially in diameter. |
| Core Idea: Chemical and Physical Basis of Life | |
| Life processes are the result of regulated chemical and physical interactions and reactions governed by the laws of physics. | This question asks the student to consider the high volume of glucose used by cells in cellular respiration, an essential life process. |

B12

**Biology Example 12:** Aatrex is an herbicide that kills some plants by binding to plastoquinone (Pq), the mobile electron carrier. Aatrex prevents Pq from accepting electrons. How would Aatrex affect oxygen (O_2_) production?

- 1. O_2_ production would **increase**. Aatrex binding to Pq would stop electron transport **increasing the need to replace electrons in photosystem II and increasing the splitting of water.**
  2. O_2_ production would **decrease**. Aatrex binding to Pq would stop electron transport **increasing the need to replace electrons in photosystem II and increasing the splitting of water.**
  3. O_2_ production would **increase**. Aatrex binding to Pq would stop electron transport **leaving photosystem II in a reduced state and eliminating the need to split water.**
  4. O_2_ production would **decrease**. Aatrex binding to Pq would stop electron transport **leaving photosystem II in a reduced state and eliminating the need to split water.** *

| CRITERIA | RATIONALE |
| --- | --- |
| Scientific Practice: Constructing Explanations and Engaging in Argument from Evidence | |
| 1. Question gives an event, observation, or phenomenon. 2. Question gives or asks student to select a claim based on the given event, observation, or phenomenon. 3. Question asks student to select scientific principles or evidence in the form of data or observations to support the claim. 4. Question asks student to select the reasoning about why the scientific principles or evidence support the claim. | 1. Question describes an observation: “Aatrex is an herbicide that kills some plants by binding to plastoquinone (Pq), the mobile electron carrier.” 2. Correct answer includes “O_2_ production would decrease”. 3. Correct answer includes “Aatrex binding to Pq would stop electron transport”. 4. Correct answer includes “leaving photosystem II in a reduced state and eliminating the need to split water”. |
| Crosscutting Concept: Cause and Effect: Mechanism and Explanation | |
| To code an assessment task with Cause and Effect: Mechanism and Explanation, the question provides at most two of the following: 1) a cause, 2) an effect, and 3) the mechanism that links the cause and effect, and the student is asked to provide the other(s). | The question provides the cause (Aatrex binds to Pq) and asks for the effect (what happens to oxygen production) and the mechanism (what happens to photosystem II). |
| Core Idea: Not Applicable | |

B13

**Biology Example 13:** Consider the following statement made by an undergraduate student. “The wood produced by an oak tree comes primarily from the nutrients and water in the soil.” Do you agree with this statement?

- 1. Yes, wood is mostly made out of carbon that comes from the ground.
  2. No, wood is mostly made out of carbon that comes from the air. *
  3. Yes, wood is mostly made out of nitrogen that comes from the ground.
  4. No, wood is mostly made out of nitrogen that comes from the air.

| CRITERIA | RATIONALE |
| --- | --- |
| Scientific Practice: Evaluating Information | |
| 1. Question gives an excerpt from a conversation, article, student solution, or video (or similar form of communication) that makes one or more assertions. 2. Question gives a conclusion about the validity of the assertion(s) or asks student to select a conclusion about the validity of the assertion(s) or reconciliation. 3. Question asks student to select reasoning to support their conclusion(s) about the validity of the assertion(s) or reconciliation with data, observations, or scientific principles. | 1. Question provides a quote from a student. 2. Question asks student to choose whether or not they agree with the student. 3. Question asks student to select the reason why they do or don’t agree with the student. |
| Crosscutting Concept: Not Applicable | |
| Core Idea: Matter and Energy | |
| Free energy and matter are utilized in regulated molecular processes that establish order, support growth and development, and regulate dynamic homeostasis in cells. | The question asks the student to identify what type of matter contributes to the bulk of mass in an oak tree, and where that mass comes from. |

## Chemistry Exemplars

C1

**Chemistry Example 1:** Given the data set below, provide at least three questions that could be answered by analyzing the data.

| Concentration X (mol/L) | Concentration Y (mol/L) | Initial rate (s^–1^) |
| --- | --- | --- |
| 2 | 2 | 2 |
| 2 | 4 | 2 |
| 4 | 4 | 8 |

| CRITERIA | RATIONALE |
| --- | --- |
| Scientific Practice: Asking Questions | |
| 1. Question gives an event, observation, phenomenon, data, scenario, or model. 2. Question asks student to generate an empirically testable question about the given event, observation, phenomenon, data, scenario, or model. | 1. Question includes a data set. 2. Question asks student to “Provide at least three questions that could be answered by analyzing the data”. |
| Crosscutting Concept: Not Applicable | |
| Core Idea: Not Applicable | |

C2

**Chemistry Example 2:** Use the Lewis structures for trimethylamine **N(CH_3_)_3_** and propylamine **CH_3_CH_2_CH_2_NH_2_** to answer the following questions:


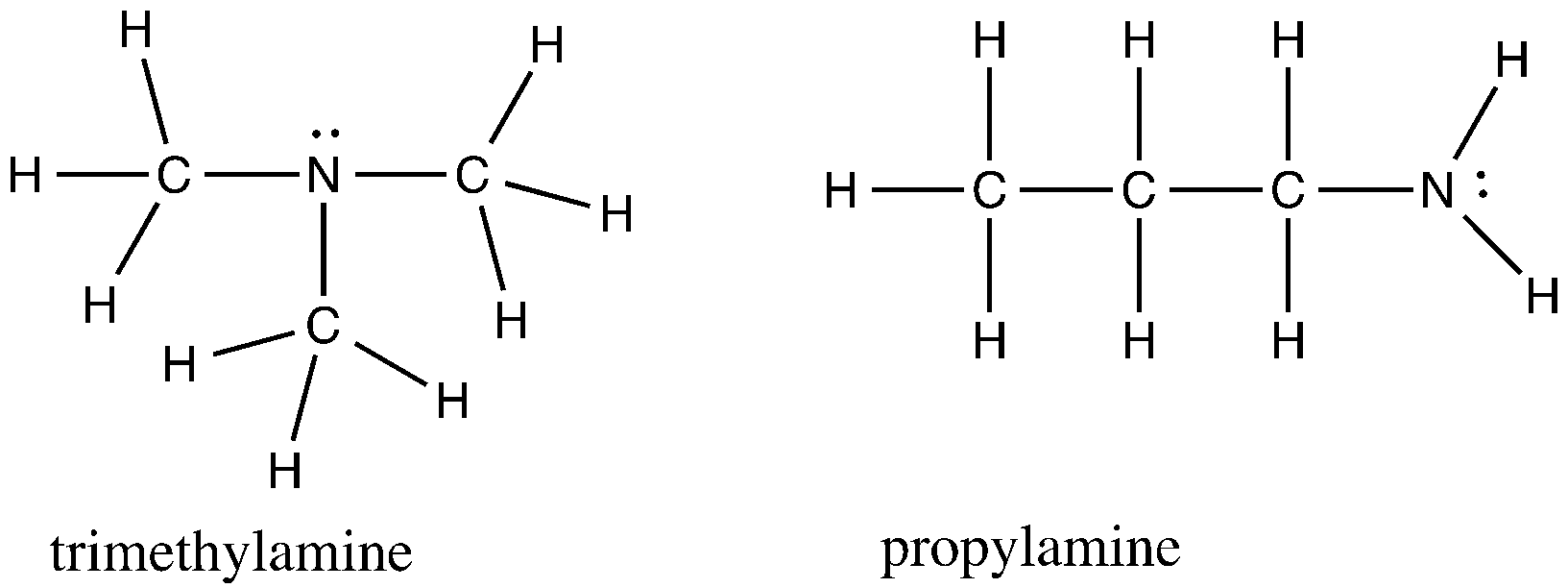


- 1. For a container of trimethylamine, what intermolecular forces would be present?
  2. For a container of propylamine, what intermolecular forces would be present?
  3. Draw three molecules of trimethylamine showing the **strongest intermolecular force (IMF)** for this compound **labeling the location and type** of IMF you are drawing**.**
  4. Draw three molecules of propylamine showing the **strongest intermolecular force** for this compound **labeling the location and type** of IMF you are drawing**.**
  5. Using the relative strengths of intermolecular forces, predict which compound would have the higher boiling point and explain why?

| CRITERIA | RATIONALE |
| --- | --- |
| Scientific Practice: Developing and Using Models | |
| 1. Question gives an event, observation, or phenomenon for the student to explain or make a prediction about. 2. Question gives a representation or asks student to construct a representation. 3. Question asks student to explain or make a prediction about the event, observation, or phenomenon. 4. Question asks student to provide the reasoning that links the representation to their explanation or prediction. | 1. Question mentions the phenomenon of “boiling point”. 2. Question gives student two Lewis structures and also asks student to construct a representation showing the intermolecular forces between trimethylamine molecules and those between propylamine molecules. 3. Question asks student to “predict which compound would have the higher boiling point”. 4. Question asks student to answer “Using the relative strengths of intermolecular forces…explain why”. |
| Crosscutting Concept: Cause and Effect: Mechanism and Explanation | |
| To code an assessment task with Cause and Effect: Mechanism and Explanation, the question provides at most two of the following: 1) a cause, 2) an effect, and 3) the mechanism that links the cause and effect, and the student is asked to provide the other(s). | The question asks the student to identify the relative strengths of intermolecular forces (cause), explain how the strengths of the intermolecular forces influence the boiling point (mechanism), and then predict which compound has the higher boiling point (effect). |
| Core Idea: Atomic/Molecular Structure and Properties | |
| The macroscopic physical and chemical properties of a substance are determined by the three-dimensional structure, the distribution of electron density, and the nature and extent of the noncovalent interactions between particles. | The question asks the student to identify as well as draw how three molecules of each substance (trimethylamine and propylamine) would interact, which means the student must first take into consideration the three-dimensional structure and distribution of electron density to correctly understand the noncovalent interactions present. The question then asks the the student to concatenate all of this information to determine how these interactions would influence the boiling point of the substances. |

C3

**Chemistry Example 3:** Design an experiment to investigate whether there is a change in mass when water melts. Describe the experimental set up, the data you would collect, and how you would analyze the data. Explain why each piece of data is needed.

| CRITERIA | RATIONALE |
| --- | --- |
| Scientific Practice: Planning Investigations | |
| 1. Question poses a scientific question, claim, or hypothesis to be investigated. 2. Question asks student to describe or design an investigation, or identify the observations required to answer the question, to answer the question or test the claim or hypothesis. 3. Question asks student to justify how their description, design, or observations can be used to answer the question or test the claim or hypothesis. | 1. Question poses a scientific question: “whether there is a change in mass when water changes phase”. 2. Question asks student to “Describe the experimental set up, the data you would collect”. 3. Question asks student “how you would analyze the data” and “Explain why each piece of data is needed.” as a justification for the design. |
| Crosscutting Concept: Energy and Matter: Flows, Cycles, and Conservation | |
| To code an assessment task with Energy and Matter: Flows, Cycles, and Conservation, the question asks the student to describe the transfer or transformation of energy or matter within or across systems, or between a system and its surroundings, with explicit recognition that energy and/or matter are conserved. | The question asks the student to design an experiment to determine if mass changes in a phase change. In order to design the experiment, student must recognize that in a phase change the energy added or removed from the system changes the size of the average kinetic of the water molecules relative to the potential energy of the interactions between the molecules while the number of water molecules does not change. |
| Core Idea: Not Applicable | |

C4

**Chemistry Example 4:** Here are the atomic radii (in pm) for the third row of the periodic table:

| Na | 157 |
| --- | --- |
| Mg | 136 |
| Al | 125 |
| Si | 117 |
| P | 110 |
| S | 104 |
| Cl | 100 |

1. Make a plot of the atomic radius (y axis) vs. atomic number (element, on the x axis)

Here is a table of the first ionization energies (energy required to remove the outermost electron) for the third row of the periodic table (in kJ/mol):

| Na | 495.8 |
| --- | --- |
| Mg | 737.7 |
| Al | 577.6 |
| Si | 786.4 |
| P | 1011.7 |
| S | 999.6 |
| Cl | 1251.1 |

2. Make a plot of the first ionization energy (y axis) vs. atomic number (element, on the x axis)

3. Compare the two graphs. In what ways are the trends in each graph related? Explain how and why they are related, including a discussion of forces and energy in your answer.

| CRITERIA | RATIONALE |
| --- | --- |
| Scientific Practice: Analyzing and Interpreting Data | |
| 1. Question gives a scientific question, claim, or hypothesis to be investigated. 2. Question gives a representation of the data (e.g., table or graph, or list of observations) provided to answer the question or test the claim or hypothesis. 3. Question gives an analysis of the data or asks student to analyze the data. 4. Question asks student to interpret the results or assess the validity of the conclusions in the context of the scientific question, claim, or hypothesis. | 1. Question poses the question “In what ways are the trends in each graph related?” 2. Question gives a set of data for atomic radii and for ionization energies. 3. Question asks student “In what ways are the trends in each graph related?” 4. Question asks student to interpret the results by asking them to “Explain how and why they are related”. |
| Crosscutting Concept: Patterns | |
| To code an assessment task with Patterns, the question asks the student to identify patterns or trends emerging from three or more events, observations, or data. | The question asks the student to plot the atomic radii and ionization energies of elements in a row of the periodic table as a function of atomic number. Then the question asks the student to identify the trends in the behavior of the atomic radii and ionization energies, the relationship between the trends for atomic radii and ionization energies, and to explain this relationship. |
| Core Idea: Energy - Quantum Mechanical Energy Levels and Changes | |
| Energy levels are quantized in atoms and molecules resulting in discrete energies for transitions between energy levels. This is a direct consequence of the wave-particle duality of electrons and other subatomic particles. | The idea of distinct energy levels means that specific amounts of energy are needed to remove an electron from the atom. In addition, the attractive forces between the protons in the nucleus and the outer electron being removed influence the amount of energy required. |

C5

**Chemistry Example 5:** Suppose that you have 10 grams of carbon and 4 grams of hydrogen available. Do you have enough starting materials to produce 14 grams of methane (CH_4_)? If yes, explain why and how much methane you can make. If no, explain why not and show the maximum amount of methane that could be prepared. **Be sure to include a balanced chemical equation and all calculations needed to support your answer to receive full credit.**

| CRITERIA | RATIONALE |
| --- | --- |
| Scientific Practice: Using Mathematics and Computational Thinking | |
| 1. Question gives an event, observation, or phenomenon. 2. Question asks student to perform a calculation or statistical test, generate a mathematical representation, or demonstrate a relationship between parameters. 3. Question asks student to give a consequence or an interpretation (not a restatement) in words, diagrams, symbols, or graphs of their results in the context of the given event, observation, or phenomenon. | 1. Question describes an event where “You have 10 grams of carbon and 4 grams of hydrogen available… to produce 14 grams of methane”. 2. Question asks student to “include all calculations” necessary to answer the question “Do you have enough starting materials to produce 14 grams of methane?”. 3. Question asks student to predict “How much methane you can make” and identify “the maximum amount of methane that could be prepared” |
| Crosscutting Concept: Energy and Matter: Flows, Cycles, and Conservation | |
| To code an assessment task with Energy and Matter: Flows, Cycles, and Conservation, the question asks the student to describe the transfer or transformation of energy or matter within or across systems, or between a system and its surroundings, with explicit recognition that energy and/or matter are conserved. | The question asks the student to use the balanced chemical equation for the reaction of carbon with hydrogen to form methane, which represents conservation of matter in the reaction, and to determine how much methane could be produced from the quantities of carbon and hydrogen specified. |
| Core Idea: Not Applicable | |

C6

**Chemistry Example 6:** The ionization of electrons from an aluminum atom requires energy.

1. Circle the graph that correctly represents the first five ionization energies of aluminum.


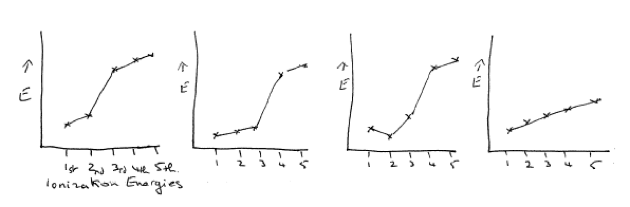


1. What information from the graph made you choose this answer (this is your evidence)?
2. Discuss why the patterns in successive ionization energies in the graph that you selected in question 1 are consistence with what you would expect for aluminum. Be sure to provide an argument that includes a discussion of both the **forces and energy** changes involved.

| CRITERIA | RATIONALE |
| --- | --- |
| Scientific Practice: Constructing Explanations and Engaging in Argument from Evidence | |
| 1. Question gives an event, observation, or phenomenon. 2. Question gives or asks student to make a claim based on the given event, observation, or phenomenon. 3. Question asks student to provide scientific principles or evidence in the form of data or observations to support the claim. 4. Question asks student to provide reasoning about why the scientific principles or evidence support the claim. | 1. Question describes a phenomenon: “ionization of electrons from an aluminum atom requires energy”. 2. Question asks student to “Circle the graph that correctly represents the first five ionization energies of aluminum.” 3. Question asks student for “What information from the graph made you choose this answer?” 4. Question asks student to “Discuss why the patterns in successive ionization energies are consistent with what you would expect for aluminum.” |
| Crosscutting Concept: Cause and Effect: Mechanism and Explanation | |
| To code an assessment task with Cause and Effect: Mechanism and Explanation, the question provides at most two of the following: 1) a cause, 2) an effect, and 3) the mechanism that links the cause and effect, and the student is asked to provide the other(s). | The question asks the student to discuss how the strength of the electrostatic forces in an atom and atomic structure (cause) impact the amount of energy required to remove successive electrons (mechanism) leading to the pattern of successive ionization energies (effect). |
| Core Idea: Energy - Quantum Mechanical Energy Levels and Changes | |
| Energy levels are quantized in atoms and molecules resulting in discrete energies for transitions between energy levels. This is a direct consequence of the wave-particle duality of electrons and other subatomic particles. | The idea of distinct energy levels means that specific amounts of energy are needed to remove an electron from the atom. In addition, the attractive forces between the protons in the nucleus and the electron being removed influence the amount of energy required. The energy needed to remove a valence electron increases with the number of electrons being removed. When a core electron is removed, substantially more energy is required because these electrons are closer to the nucleus on average and experience less shielding causing a jump in the ionization energy. |

C7

**Chemistry Example 7:** The following drawing was taken from a student response on a previous short answer exam question.

Original question:

*Consider a beaker of ice and water sitting on a counter. Draw a molecular level picture of the mixture, clearly showing the solid and liquid water and the interaction between them.*

Student response:


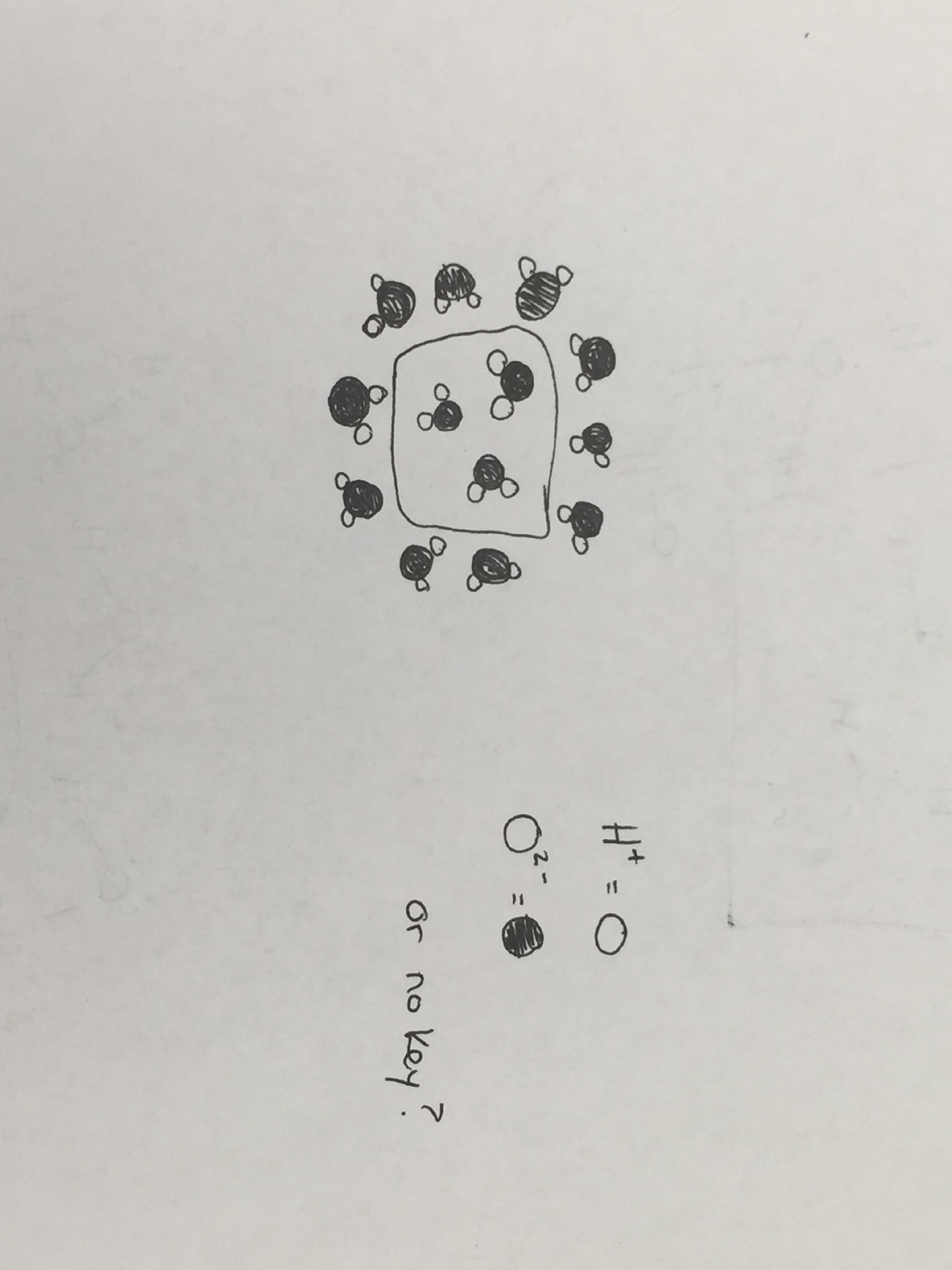


Identify which aspects of this representation are scientifically incorrect and explain why they are incorrect. Prepare a revised response that retains correct ideas and corrects errors in the previous response.

| CRITERIA | RATIONALE |
| --- | --- |
| Scientific Practice: Evaluating Information | |
| 1. Question gives an excerpt from a conversation, article, student solution, or video (or similar form of communication) that makes one or more assertions. 2. Question gives a conclusion about the validity of the assertion(s) made or asks student to make a conclusion about the validity of the assertion(s) or reconcile multiple assertions with each other. 3. Question asks student to provide reasoning to support their conclusion(s) about the validity of the assertion(s) or reconciliation with data, observations, or scientific principles. | 1. Question gives a student response to a previous exam question. 2. Question implies that the previous answer is incorrect: “Identify which aspects of this representation are scientifically incorrect.” 3. Question asks student to “explain why they are incorrect. Prepare a revised response that retains correct ideas and corrects errors in the previous response.” |
| Crosscutting Concept: Systems and System Models | |
| To code an assessment task with Systems and System Models, the question asks the student to identify a system (by defining its components or boundaries), any assumptions made, and the surroundings (if necessary), and how the system and surroundings interact with each other. | The question asks the student to prepare a revised molecular-level representation of ice (system) and liquid water (surroundings) and then illustrate and describe how the system and surroundings interact. |
| Core Idea: Electrostatic and Bonding Interactions | |
| Attractive and repulsive electrostatic forces govern noncovalent and bonding (covalent and ionic) interactions between atoms and molecules. The strength of these forces depends on the magnitude of the charges involved and the distances between them. | The question asks the student to represent the electrostatic interactions (i.e. intermolecular forces) between the water molecules in the liquid phase and compare them with those in the solid phase, and those between the solid and liquid phase. |

C8


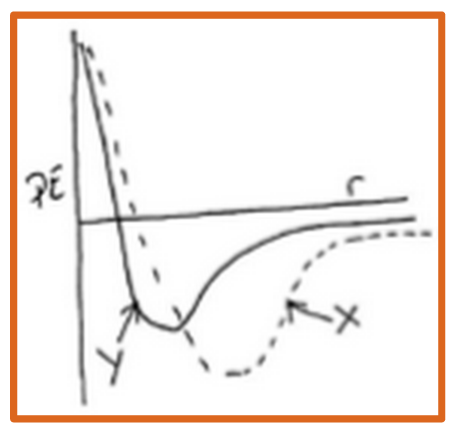


**Chemistry Example 8:** In the graph provided that shows the potential energy change as two particles of a substance approach each other, predict which curve would represent the substance with the higher boiling point and why?

- 1. X because the particles interacting must be heavier, therefore they are harder to move into the gas phase.
  2. X because the potential well is deeper, therefore more energy must be added to separate the particles. *
  3. Y because the particles are smaller and fit together better, therefore more energy must be added to separate the particles.
  4. Y because the atoms are lighter, therefore they should be easier to move around.

| CRITERIA | RATIONALE |
| --- | --- |
| Scientific Practice: Developing and Using Models | |
| 1. Question gives an event, observation, or phenomenon for the student to explain or make a prediction about. 2. Question gives student a representation or asks student to select a representation. 3. Question asks student to select an explanation for or prediction about the event, observation, or phenomenon. 4. Question asks student to select the reasoning that links the representation to their explanation or prediction. | 1. Question refers to “the substance with the highest boiling point”. 2. Question provides a graph showing potential energy curves for two substances. 3. Question asks student to “predict which curve would represent the substance with the higher boiling point”. 4. Question asks student to select reasoning that answers the question “why”. |
| Crosscutting Concept: Cause and Effect: Mechanism and Explanation | |
| To code an assessment task with Cause and Effect: Mechanism and Explanation, the question provides at most two of the following: 1) a cause, 2) an effect, and 3) the mechanism that links the cause and effect, and the student is asked to provide the other(s). | The question asks the student to identify the effect (which compound has the higher boiling point), the underlying cause (a deeper potential well meaning that the force of attraction between the particles is larger), and mechanism (more energy is needed to overcome the stronger attractive forces). |
| Core Idea: Energy - Atomic/Molecular | |
| Kinetic and potential energy changes occur when atoms and molecules interact. Energy is released to the surroundings when bonds or attractive noncovalent interactions form, and conversely energy is required to break bonds or noncovalent interactions. | The question gives the student a representation of how the potential energy of the system of particles changes with respect to the distance between them. The student must take into consideration that difference in energy between the minimum in potential energy (y axis) and energy of the separated particles corresponds to the amount of kinetic energy required to overcome the attractive force between the particles (i.e. boiling point). |

C9

**Chemistry Example 9:** Nitrogen dioxide and carbon monoxide react to form nitric oxide and carbon dioxide.

NO_2_(g) + CO(g) → NO(g) + CO_2_(g)

1. The order of the reaction with respect to NO_2_ could be determined by measuring the rate of this reaction while

1. holding the concentration of CO constant
2. holding the concentration of NO_2_ constant
3. doubling the concentration of CO
4. tripling concentration of NO_2_
   1. I and II
   2. I and IV *
   3. II and III
   4. III and IV

2. Which of the following statements support your choice of experimental conditions for the two measurements used to determine the reaction order with respect to NO_2_?

1. The reaction rate is determined by both reactants; therefore, it is necessary to change the concentrations of both reactants to determine the order with respect to NO_2_.
2. It is necessary to double the concentration of one of the reactants to determine the reaction order.
3. Changing the concentrations of NO_2_ and CO for the two measurements makes it impossible to determine the effect of either reactant on the reaction rate; therefore, only one concentration should be changed while the other is held constant.
4. The order of a reaction with respect to a reactant describes how the reaction rate depends on the concentration of that particular reactant.
   1. I
   2. I and II
   3. III and IV *
   4. IV

| CRITERIA | RATIONALE |
| --- | --- |
| Scientific Practice: Planning Investigations | |
| 1. Question poses a scientific question, claim, or a hypothesis to be investigated. 2. Question asks student to select a description of or a design for an investigation or select the observations that could be used to answer the question or test the hypothesis. 3. Question asks student to select a justification of how the description, design, or observations can be used to answer the question or test the hypothesis. | 1. Question asks student a question about how “the order of the reaction with respect to NO_2_” could be determined. 2. Correct answer includes “I. Hold concentration of CO constant” and “IV. Triple the concentration of NO_2_”. 3. Correct answer includes “III. Changing the concentrations of NO_2_ and CO for the two measurements makes it impossible to determine the effect of either reactant on the reaction rate; therefore, only one concentration should be changed while the other is held constant.” and “IV. The order of a reaction with respect to a reactant describes how the reaction rate depends on the concentration of that particular reactant.” |
| Crosscutting Concept: Proportion and Quantity | |
| To code an assessment task with Proportion and Quantity, the question asks the student to predict the response of one variable to changes in another or identify the relationship between two or more variables from data. | The questions requires the student to recognize that reaction rates are proportional to reactant concentrations raised to some power (order) and that the concentration of CO must be held constant while changing the concentration of NO_2_ in order to identify the rate of reaction. |
| Core Idea: Not Applicable | |

C10

**Chemistry Example 10:** The graph below represents the temperature change vs. time when thermal energy is added at a constant rate to two equal masses of copper (Cu) (specific heat 0.385 J/g K) and aluminum (Al) (specific heat 0.903 J/g K). Which metal does the dotted line correspond to, and why?


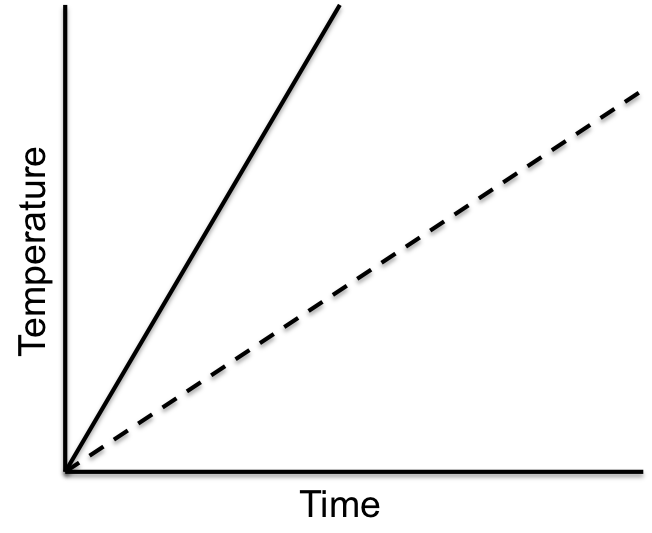


1. Cu, because it has the lower specific heat and will take longer to heat up.
2. Cu, because it has the lower specific heat and will heat up faster.
3. Al, because it has the higher specific heat and will take longer to heat up. *
4. Al, because it has the higher specific heat and will heat up faster.

| CRITERIA | RATIONALE |
| --- | --- |
| Scientific Practice: Analyzing and Interpreting Data | |
| 1. Question gives a scientific question, claim, or a hypothesis to be investigated. 2. Question gives a representation of data (table, graph, list of observations, etc.) provided to answer the question or test the claim or hypothesis. 3. Question asks student to select an interpretation of the results or an assessment of the validity of the conclusions in the context of the scientific question, claim, or hypothesis. | 1. Question asks, “Which metal does the dotted line correspond to?” 2. Question gives a “temperature change vs. time” graph. 3. Question asks student to make a claim about which metal the data represented by the dotted line corresponds to and select a justification for this interpretation of the data. |
| Crosscutting Concept: Energy and Matter: Flows, Cycles, and Conservation | |
| To code an assessment task with Energy and Matter: Flows, Cycles, and Conservation, the question asks the student to describe the transfer or transformation of energy or matter within or across systems, or between a system and its surroundings, with explicit recognition that energy and/or matter are conserved. | The question asks the student to identify that more heat is needed increase the temperature of Al because of its higher specific heat. |
| Core Idea: Energy - Macroscopic | |
| Changes in phase and reactions of collections of atoms and/or molecules are accompanied by energy changes that result from energy changes on the atomic/molecular scale. Temperature is determined by the average kinetic energy of collections of atoms and/or molecules. | The rate of the change in temperature (macroscopic property) corresponds to the amount of thermal energy added to the system. When thermal energy is added to two different systems at a constant rate, the specific heat (amount of energy needed to raise the temperature one degree per mass unit) can be determined. |

C11

**Chemistry Example 11:** The wavelength of an electron traveling at 1% of the speed of light is closest in size to?

1. The height of Mt. Everest *
2. The height of a person
3. The diameter of a cell in your body
4. The distance between two atoms bonded together

| CRITERIA | RATIONALE |
| --- | --- |
| Scientific Practice: Using Mathematics and Computational Thinking | |
| 1. Question gives an event, observation, or phenomenon. 2. Question asks student to perform a calculation or statistical test, use a mathematical representation, or derive a relationship between parameters in order to obtain the correct answer. 3. Question asks student to select a consequence or an interpretation (not a restatement) in words, diagrams, symbols, or graphs of their results in the context of the given event, observation, or phenomenon. | 1. Question describes an event where “an electron [is] traveling at 1% of the speed of light” 2. Question requires calculation (or approximation) of the de Broglie wavelength for an electron. 3. Question requires student to relate the number calculated to the size of a physical object. |
| Crosscutting Concept: Scale | |
| To code an assessment task with Scale, the question asks the student 1) to compare objects, processes, or properties across size, time, or energy scales, or to dimensions of familiar objects, timescales, or energies or 2) to identify non-negligible/relevant interactions at various scales. | The question asks the student to compare the calculated value for the de Broglie wavelength of an electron traveling at 1% of the speed of light to objects with dimensions on different scales. |
| Core Idea: Not Applicable | |

C12

Chemistry Example 12: One of these two compounds is a liquid at room temperature. Choose the compound, the evidence you are using to make the claim, and the reasoning that allows you to make this claim.

Compound:


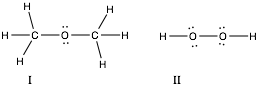


Evidence:

III. Compound I is heavier than compound II.

IV. Compound I has more hydrogens and can form more hydrogen bonds than II.

V. Compound II has both hydrogens and oxygens capable of hydrogen bonding.

Reasoning:

VI. Heavier molecules are more likely to cluster together and form liquids because they are attracted to each other strongly by London dispersion forces.

VII. Molecules capable of hydrogen bonding are strongly attracted to each other and tend to cluster together to form liquids.

1. I, III, VI
2. I, IV, VII
3. II, V, VII *
4. Not enough information

| CRITERIA | RATIONALE |
| --- | --- |
| Scientific Practice: Constructing Explanations and Engaging in Argument from Evidence | |
| 1. Question gives an event, observation, or phenomenon. 2. Question gives or asks student to select a claim based on the given event, observation, or phenomenon. 3. Question asks student to select scientific principles or evidence in the form of data or observations to support the claim. 4. Question asks student to select the reasoning about why the scientific principles or evidence support the claim. | 1. Question asks about a phenomenon: a compound is a “liquid at room temperature”. 2. Question asks student to select a “compound”. 3. Question asks student to select “evidence”. 4. Question asks student to select “reasoning”. |
| Crosscutting Concept: Cause and Effect: Mechanism and Explanation | |
| To code an assessment task with Cause and Effect: Mechanism and Explanation, the question provides at most two of the following: 1) a cause, 2) an effect, and 3) the mechanism that links the cause and effect, and the student is asked to provide the other(s). | The question asks the student to identify the effect (which compound is a liquid at room temperature), the underlying cause (type of intermolecular forces available), and mechanism (stronger interactions between molecules). |
| Core Idea: Electrostatic and Bonding Interactions | |
| Attractive and repulsive electrostatic forces govern noncovalent and bonding (covalent and ionic) interactions between atoms and molecules. The strength of these forces depends on the magnitude of the charges involved and the distances between them. | The question asks the student to recognize that Compound 2 is able to participate in hydrogen bonding while Compound 1 cannot. The ability for hydrogen bonding between the molecules in Compound 2 results in stronger attractive forces. |

C13

**Chemistry Example 13:** A website for natural, organic products states that “organic Vitamin C isolated from citrus fruit is superior to Vitamin C made in a laboratory”. Do you agree and why?

A. Yes, Vitamin C made in a laboratory has chemicals in it.

B. Yes, natural compounds are always better than laboratory made chemicals.

C. No, the properties of Vitamin C are determined by its structure, not by its origin. *

D. No, the body can rid itself of the impurities in laboratory made Vitamin C.

| CRITERIA | RATIONALE |
| --- | --- |
| Scientific Practice: Evaluating Information | |
| 1. Question gives an excerpt from a conversation, article, student solution, or video (or similar form of communication) that makes one or more assertions. 2. Question gives a conclusion about the validity of the assertion(s) or asks student to select a conclusion about the validity of the assertion(s) or reconciliation. 3. Question asks student to select reasoning to support their conclusion(s) about the validity of the assertion(s) or reconciliation with data, observations, or scientific principles. | 1. Question presents the student with the statement that “organic Vitamin C isolated from citrus fruit is superior to Vitamin C made in a laboratory”. 2. Question asks the student to select whether or not they agree with the statement provided. 3. Question asks the student to select the reasoning that supports why they do or do not agree with the statement provided. |
| Crosscutting Concept: Structure and Function | |
| To code an assessment task with Structure and Function, the question asks the student to predict or explain a function or property based on a structure, or to describe what structure could lead to a given function or property. | This question asks the student to recognize that it is the structure of a compound that predicts its macroscopic properties and not whether a compound is extracted naturally or made synthetically in the laboratory. |
| Core Idea: Atomic/Molecular Structure and Properties | |
| The macroscopic physical and chemical properties of a substance are determined by the three-dimensional structure, the distribution of electron density, and the nature and extent of the noncovalent interactions between particles. | This question asks the student to recognize that it is the structure of a compound that predicts its macroscopic properties and not whether a compound is extracted naturally or made synthetically in the laboratory. |

## Physics Exemplars

P1

**Physics Example 1:** You have worked on the Voyager 1 project for a number of years. You receive a satellite phone call while on a team building exercise with your fellow scientists that there is an emergency with Voyager 1. You must escape hostile territory while being pursued by an enemy who wants you for your scientific prowess. You come to a river that is fast flowing, and the only safe landing point on the opposite bank is directly across from an abandoned but functional boat. The width of the river is 1.8 km. The boat has a constant speed of 21 knots. However, the boat’s rudder has been damaged so the boat cannot be redirected once it has started moving. With you, you have a remote controlled boat, which travels at a constant speed, but you do not know what that speed is. You also have a stopwatch.

What testable questions do you need to answer in order to make it safely to the landing point?

| CRITERIA | RATIONALE |
| --- | --- |
| Scientific Practice: Asking Questions | |
| 1. Question gives an event, observation, phenomenon, data, scenario, or model. 2. Question asks student to generate an empirically testable question about the given event, observation, phenomenon, data, scenario, or model. | 1. Question describes a scenario in which the student is positioned at the edge of a river they must cross. 2. Question asks student “What testable questions do you need to answer in order to make it safely to the landing point?”. |
| Crosscutting Concept: Not Applicable | |
| Core Idea: Not Applicable | |

P2

**Physics Example 2:** A woman is trying to load a piano (~200 kg) up a ramp into a pickup truck. She can exert a pushing force of about 800 N. The ramp is inclined at an angle of ~10° above the horizontal. Will she be able to push the piano up the ramp? If the answer is yes, determine the maximum acceleration the piano can have. If the answer is no, suggest a potential change that could be made to the system/situation that would allow the woman to push the piano up the ramp. Justify your answers with words, a free body diagram, and equations where necessary.

| CRITERIA | RATIONALE |
| --- | --- |
| Scientific Practice: Developing and Using Models | |
| 1. Question gives an event, observation, or phenomenon for the student to explain or make a prediction about. 2. Question gives a representation or asks student to construct a representation. 3. Question asks student to explain or make a prediction about the event, observation, or phenomenon. 4. Question asks student to provide the reasoning that links the representation to their explanation or prediction. | 1. Question is about an event where “A woman is trying to load a piano”. 2. Question asks student to generate “A free body diagram, and equations”. 3. Question asks student to predict: “Will she be able to push the piano up the ramp?” 4. Questions asks student to “Justify your answers with words, a free body diagram, and equations where necessary.” |
| Crosscutting Concept: Systems and System Models | |
| To code an assessment task with Systems and System Models, the question asks the student to identify a system (by defining its components or boundaries), any assumptions made, and the surroundings (if necessary), and how the system and surroundings interact with each other. | Question asks the student to justify their answer with words and a free body diagram. |
| Core Idea: Interactions Can Cause Changes in Motion | |
| Changes in an object’s motion are the result of interactions between it and one or more other objects. | Question involves a woman who is interacting with the piano, causing a change in the piano’s motion. |

P3

**Physics Example 3:** Design an experiment, including the measurements needed, to test the claim that the current is the same everywhere in a series circuit. Explain the rationale for your design, including any underlying assumptions you’ve made.

| CRITERIA | RATIONALE |
| --- | --- |
| Scientific Practice: Planning Investigations | |
| 1. Question poses a scientific question, claim, or hypothesis to be investigated. 2. Question asks student to describe or design an investigation, or identify the observations required to answer the question, to answer the question or test the claim or hypothesis. 3. Question asks student to justify how their description, design, or observations can be used to answer the question or test the claim or hypothesis. | 1. Question makes the claim that “the current is the same everywhere in a series circuit”. 2. Questions asks student to “Design an experiment”. 3. Question asks student to “Explain the rationale for your design.” |
| Crosscutting Concept: Not Applicable | |
| Core Idea: Interactions are Mediated by Fields | |
| Fields are generated by charges/masses. Fields affect charges/masses. In circuits, fields induce currents. | Question asks student to identify the measurements needed to evaluate the current everywhere in a circuit. |

P4

**Physics Example 4:** Your friend is traveling in a hovercraft across the Arctic ice and you are trying to catch them. You can’t see them, but you do have a connection with their GPS locator. Using a stopwatch, you gather the following data:

| Time (s) | Their position (m) | Your position (m) |
| --- | --- | --- |
| 10 | 536 | 10 |
| 20 | 1072 | 41 |
| 30 | 1609 | 94 |

When would you expect to catch up to your friend? Be sure to explain how you determined the time that you found and determine whether or not it is a reasonable prediction.

| CRITERIA | RATIONALE |
| --- | --- |
| Scientific Practice: Analyzing and Interpreting Data | |
| 1. Question gives a scientific question, claim, or hypothesis to be investigated. 2. Question gives a representation of the data (e.g., table or graph, or list of observations) provided to answer the question or test the claim or hypothesis. 3. Question gives an analysis of the data or asks student to analyze the data. 4. Question asks student to interpret the results or assess the validity of the conclusions in the context of the scientific question, claim, or hypothesis. | 1. Question asks student “When would you expect to catch up to your friend?” 2. Question gives two sets of position data and the corresponding times. 3. Question asks student “to explain how you determined the time that you found”. 4. Question asks student “When would you expect to catch up to your friend?” and “determine whether or not it is a reasonable prediction.” |
| Crosscutting Concept: Patterns | |
| To code an assessment task with Patterns, the question asks the student to identify patterns or trends emerging from three or more events, observations, or data. | Question gives two sets of position data for given times and asks the student to extrapolate the data to make a prediction for a later time. |
| Core Idea: Not Applicable | |

P5

**Physics Example 5:** A parallel plate capacitor is connected to a battery and has its two plates pulled further apart from each other. What happens to the amount of charge stored in the capacitor? Be sure to indicate how you found your answer.

| CRITERIA | RATIONALE |
| --- | --- |
| Scientific Practice: Using Mathematics and Computational Thinking | |
| 1. Question gives an event, observation, or phenomenon. 2. Question asks student to perform a calculation or statistical test, generate a mathematical representation, or demonstrate a relationship between parameters. 3. Question asks student to give a consequence or an interpretation (not a restatement) in words, diagrams, symbols, or graphs of their results in the context of the given event, observation, or phenomenon. | 1. Question describes an event where “A parallel plate capacitor is connected to a battery and has its two plates pulled further apart from each other”. 2. Question asks student to “indicate how you found your answer.” 3. Question asks student to interpret “What happens to the amount of charge stored in the capacitor?” |
| Crosscutting Concept: Proportion and Quantity | |
| To code an assessment task with Proportion and Quantity, the question asks the student to predict the response of one variable to changes in another or identify the relationship between two or more variables from data. | Question asks student to predict the response of charge stored to changes in distance between the plates of a capacitor. |
| Core Idea: Interactions are Mediated by Fields | |
| Fields are generated by charges/masses. Fields affect charges/masses. In circuits, fields induce currents. | Question includes changes to the capacitance of and charge stored in a capacitor while the voltage remains constant. |

P6

**Physics Example 6:** A steel cable is hung from the ceiling. A mass is attached to the end of the cable, which causes it to stretch. Upon removal of the mass, the cable returns to its original length. From an atomic perspective, explain how a steel cable can (under certain conditions) be stretched and then return to its original length.

| CRITERIA | RATIONALE |
| --- | --- |
| Scientific Practice: Constructing Explanations and Engaging in Argument from Evidence | |
| 1. Question gives an event, observation, or phenomenon. 2. Question gives or asks student to make a claim based on the given event, observation, or phenomenon. 3. Question asks student to provide scientific principles or evidence in the form of data or observations to support the claim. 4. Question asks student to provide reasoning about why the scientific principles or evidence support the claim. | 1. Question mentions an observation that “A mass is attached to the end of the cable, which causes it to stretch. Upon removal of the mass, the cable returns to its original length.” 2. Question makes a claim that “a steel cable can be stretched and return to its original length”. 3. Question asks the student to provide scientific principles to support their claim: “how a steel cable can (under certain conditions) be stretched and then return to its original length”. 4. Question asks student to link the atomic model to macroscopic observations: “explain”. |
| Crosscutting Concept: Structure and Function | |
| To code an assessment task with Structure and Function, the question asks the student to predict or explain a function or property based on a structure, or to describe what structure could lead to a given function or property. | Question asks student to explain the property that a steel cable can stretch based on its atomic structure. |
| Core Idea: Interactions Can Cause Changes in Motion | |
| Changes in an object’s motion are the result of interactions between it and one or more other objects. | Question involves the interaction of a mass and a steel cable, as well as the interaction between atoms in the cable. |

P7

**Physics Example 7:** Consider the following conversation between two biology students:

Liz: I work in a microbiology lab and in our experiments we are often manipulating the motion of objects suspended in a fluid. To get these objects to move at a constant speed, we have to apply a force that is proportional to the velocity of the object.

Jack: That can’t be right. In physics, I learned that force is equal to the mass of the object times the acceleration of the object.

Are the perspectives of these two students consistent or contradictory? If consistent, explain how they can both make sense simultaneously. If contradictory, identify which is more correct and explain how the other one’s statement is wrong.

| CRITERIA | RATIONALE |
| --- | --- |
| Scientific Practice: Evaluating Information | |
| 1. Question gives an excerpt from a conversation, article, student solution, or video (or similar form of communication) that makes one or more assertions. 2. Question gives a conclusion about the validity of the assertion(s) made or asks student to make a conclusion about the validity of the assertion(s) or reconcile multiple assertions with each other. 3. Question asks student to provide reasoning to support their conclusion(s) about the validity of the assertion(s) or reconciliation with data, observations, or scientific principles. | 1. Question gives an excerpt from “the following conversation between two biology students”. 2. Question asks student to reconcile the assertions: “Are the perspectives of these two students consistent or contradictory?” 3. Question asks student to “explain how they can both make sense simultaneously.” |
| Crosscutting Concept: Cause and Effect: Mechanism and Explanation | |
| To code an assessment task with Cause and Effect: Mechanism and Explanation, the question provides at most two of the following: 1) a cause, 2) an effect, and 3) the mechanism that links the cause and effect, and the student is asked to provide the other(s). | Question gives a cause (applying a force proportional to velocity) and an effect (moving at constant speed) and asks student to make sense of the mechanism between the two (hopefully making a distinction between force and net force).  Question describes how the student applies “a force that is proportional to the velocity of the object” |
| Core Idea: Interactions Can Cause Changes in Motion | |
| Changes in an object’s motion are the result of interactions between it and one or more other objects. Multiple interactions between an object and its surroundings can result in a predictable change in motion. | Question involves the forces acting on objects suspended in a fluid and their resulting motion. |

P8
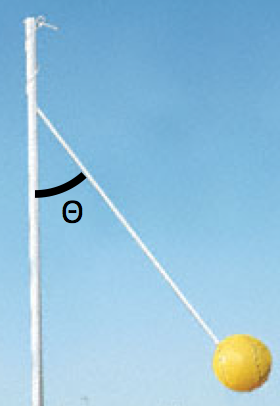


**Physics Example 8:** You are watching two of your friends play tetherball, and you notice that the faster the ball moves, the larger the angle the rope makes with the pole. In order to figure out why, you begin by thinking about the forces involved.

1. Which of the following free body diagrams would apply to the ball swinging freely around the pole?

- 1. *

1. Which of the following equations can be extracted from the correct free body diagram above (T is the tension in the rope; r is the distance between the ball and the pole; v is the speed of the ball)?
   1. $T sin \theta=\frac{m v^{2}}{r}$ *
   2. $Tcos \theta=\frac{{m v}^{2}}{r}$
   3. $T sin \theta=0$
   4. $T cos \theta=0$
2. Combine the equations you obtained from the free body diagram above, and solve for θ. Which of the following explains why the angle increases?
   1. As the speed increases, the angle must increase in order for the net force to maintain the ball’s circular trajectory. *
   2. As the ball speeds up, the radius decreases and the angle must increase in order for the net force to maintain the ball’s circular trajectory.
   3. The faster the ball moves, the larger the net force pushes outward on the ball causing it to increase the angle it makes with the vertical.

| CRITERIA | RATIONALE |
| --- | --- |
| Scientific Practice: Developing and Using Models | |
| 1. Question gives an event, observation, or phenomenon for the student to explain or make a prediction about. 2. Question gives student a representation or asks student to select a representation. 3. Question asks student to select an explanation for or prediction about the event, observation, or phenomenon. 4. Question asks student to select the reasoning that links the representation to their explanation or prediction. | 1. Question makes the observation that “the faster the ball moves, the larger the angle the rope makes with the pole”. 2. Question asks student to select “Which of the following free body diagrams” and “Which of the following equations”. 3. Correct answer contains “As the speed increases, the angle must increase”. 4. Correct answer contains “in order for the net force to maintain the ball’s circular trajectory.” |
| Crosscutting Concept: Systems and System Models | |
| To code an assessment task with Systems and System Models, the question asks the student to identify a system (by defining its components or boundaries), assumptions, and (if necessary) the surroundings, and how they interact with each other. | Question asks student to identify the system and interactions with the surroundings (free-body diagram). |
| Core Idea: Interactions Can Cause Changes in Motion | |
| Changes in an object’s motion are the result of interactions between it and one or more other objects. | Question involves the forces acting on a tetherball and how they are related to its motion. |

P9

**Physics Example 9:**

1. You want to determine the resistance of R. Which of the following circuits provided below will give you the measurements you need to determine R. Select *all* that apply.


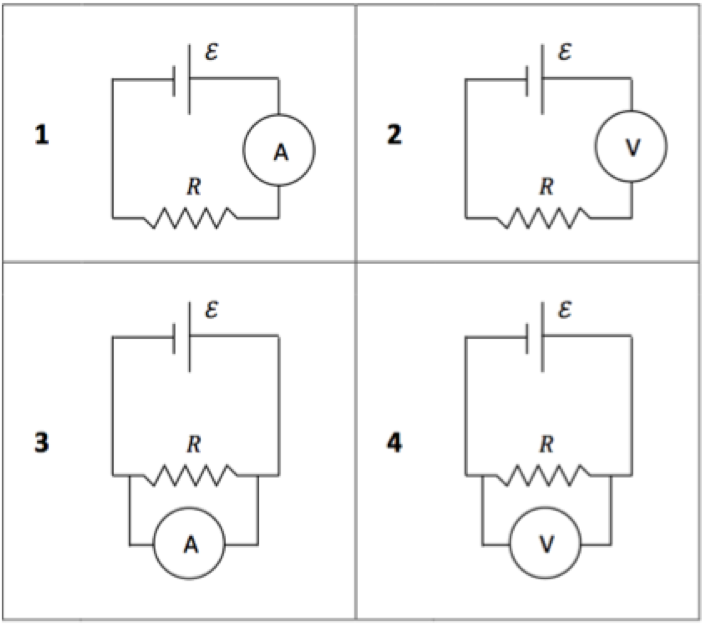


- 1. 1 *
  2. 2
  3. 3
  4. 4 *

1. Why are those measurements necessary to determine the resistance of R?
   1. We need to measure the current and voltage before the resistor for Ohm’s Law.
   2. We need to measure that the current is the same before and after the resistor.
   3. We need to measure the voltage before the resistor and current through the resistor for Ohm’s Law.
   4. We need to measure the current in the circuit and voltage across the resistor for Ohm’s Law. *

| CRITERIA | RATIONALE |
| --- | --- |
| Scientific Practice: Planning Investigations | |
| 1. Question poses a scientific question, claim, or a hypothesis to be investigated. 2. Question asks student to select a description of or a design for an investigation or select the observations that could be used to answer the question or test the hypothesis. 3. Question asks student to select a justification of how the description, design, or observations can be used to answer the question or test the hypothesis. | 1. Question asks student to “determine the resistance of R.” 2. Question asks student to select the appropriate measurements to set up within the circuit. 3. Correct answer includes the justification “because we need to know the current in the circuit and voltage across the resistor for Ohm’s Law”. |
| Crosscutting Concept: Not Applicable | |
| Core Idea: Interactions are Mediated by Fields | |
| Fields are generated by charges/masses. Fields affect charges/masses. In circuits, fields induce currents. | Question asks about the measurements needed to calculate the resistance, one of which is the voltage across the resistor. |

P10

**Physics Example 10:** You are working in a physics lab, building a circuit that will be used in a larger measurement apparatus. You are testing each piece of your circuit to determine if you need to replace it or not and collect the following I-V measurements for the branch shown:


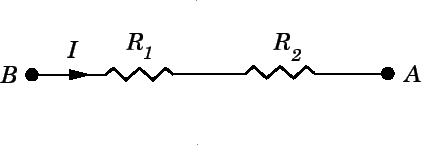


| Potential Difference between A and B (V) | Current through branch (I) |
| --- | --- |
| 0.5 | 0.3 |
| 1.0 | 0.6 |
| 1.5 | 0.9 |
| 2.0 | 1.5 |
| 2.5 | 2.1 |

Based on these data, what can you conclude about this branch of the circuit (select all that apply)?

1. The power limit for this circuit is 5.25 W.
2. The power limit for this circuit is 1.35 W. *
3. Both resistors appear to fail; they both have reached their power limits.
4. One resistor appears to fail; it has reached its power limit.
5. From these data, I cannot tell which resistor has failed. *
6. This branch of the circuit obeys Ohm’s Law for all measurements.
7. This branch of the circuit obeys Ohm’s Law up to 1.5V. *
8. This branch of the circuit should be remade. *
9. This branch of the circuit is fine and does not need to be remade.

| CRITERIA | RATIONALE |
| --- | --- |
| Scientific Practice: Analyzing and Interpreting Data | |
| 1. Question gives a scientific question, claim, or a hypothesis to be investigated. 2. Question gives a representation of data (table, graph, list of observations, etc.) provided to answer the question or test the claim or hypothesis. 3. Question asks student to select an interpretation of the results or an assessment of the validity of the conclusions in the context of the scientific question, claim, or hypothesis. | 1. Question asks student to “determine if you need to replace [the resistor(s)] or not”. 2. Data about potential differences and current are presented in a table. 3. Correct answer includes “B) The power limit for this circuit is 1.35 W.”, “E) From these data, I cannot tell which resistor has failed.”, “G) This branch of the circuit obeys Ohm’s Law up to 1.5V.”, and “H) This branch of the circuit should be remade.” |
| Crosscutting Concept: Patterns | |
| To code an assessment task with Patterns, the question asks the student to identify patterns or trends emerging from three or more events, observations, or data. | Question gives data on “Potential Difference between A and B (V)” and “Current through branch (I)” and asks the student to make conclusions based on the patterns in the data. |
| Core Idea: Interactions are Mediated by Fields | |
| Fields are generated by charges/masses. Fields affect charges/masses. In circuits, fields induce currents. | Question gives a set of data that includes voltage and current, asking the student to interpret that data. |

P11

**Physics Example 11:**

1. A parallel plate capacitor is connected to a battery and has its two plates pulled further apart from each other. What happens to the charge stored in the capacitor?
   1. increases
   2. decreases *
   3. stays the same
2. Which of the following options helps back up your answer (select all that apply)?
   1. Pulling the plates apart increases the capacitance.
   2. Pulling the plates apart decreases the capacitance. *
   3. The charge on the capacitor is held constant by the battery.
   4. The potential across the capacitor is held constant by the battery. *
   5. Q = C V *
   6. V = I R
   7. C = **Κ** C_0_

| CRITERIA | RATIONALE |
| --- | --- |
| Scientific Practice: Using Mathematics and Computational Thinking | |
| 1. Question gives an event, observation, or phenomenon. 2. Question asks student to perform a calculation or statistical test, use a mathematical representation, or derive a relationship between parameters in order to obtain the correct answer. 3. Question asks student to select a consequence or an interpretation (not a restatement) in words, diagrams, symbols, or graphs of their results in the context of the given event, observation, or phenomenon. | 1. Question describes an event where “A parallel plate capacitor is connected to a battery and has its two plates pulled further apart from each other.” 2. Question asks student “Which of the following options helps back up your answer?” 3. Question asks student to determine “What happens to the charge stored in the capacitor?” |
| Crosscutting Concept: Proportion and Quantity | |
| To code an assessment task with Proportion and Quantity, the question asks the student to predict the response of one variable to changes in another or identify the relationship between two or more variables from data. | Question asks student to predict the response of charge stored to changes in the distance between plates. |
| Core Idea: Interactions are Mediated by Fields | |
| Fields are generated by charges/masses. Fields affect charges/masses. In circuits, fields induce currents. | Question asks student about changes in charge on a capacitor and steps in their reasoning for it, which includes statements about the potential across the capacitor |

P12

**Physics Example 12:** Two identical conducting balls, A and B, are attached to insulating stands. The net charge on ball A is +Q_0_, and there is no net charge on ball B. Balls A and B are observed to attract.


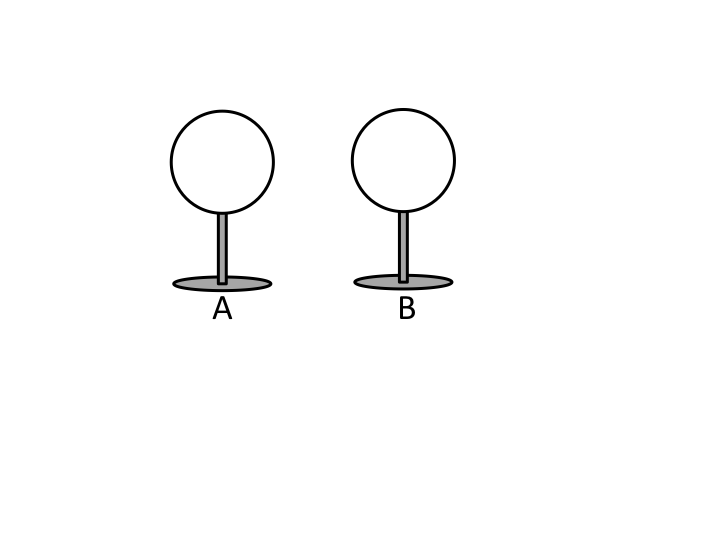


Which of the following is the best explanation for this observation?

- 1. The positive net charge on ball A attracts the electrons in ball B more than it repels the protons in ball B because the electrons are less massive than the protons, leading to a larger force on the electrons.
  2. The electrons in ball B rearrange themselves to be closer to ball A because the electrons are attracted to the positive charge in ball A and are free to move in the metal. This results in a larger attractive force on the electrons in ball B than the repulsive force on ball B’s protons. *
  3. There must actually be a small, negative net charge on ball B, which we cannot measure, because positive charges only attract negative charges and Coulomb’s law states that the net force is zero if there is no net charge.

| CRITERIA | RATIONALE |
| --- | --- |
| Scientific Practice: Constructing Explanations and Engaging in Argument from Evidence | |
| 1. Question gives an event, observation, or phenomenon. 2. Question gives or asks student to select a claim based on the given event, observation, or phenomenon. 3. Question asks student to select scientific principles or evidence in the form of data or observations to support the claim. 4. Question asks student to select the reasoning about why the scientific principles or evidence support the claim. | 1. Question describes the observation that “Balls A and B attract”. 2. Correct answer includes the claim that “The electrons in ball B rearrange themselves to be closer to ball A.” 3. Correct answer includes “electrons are attracted to the positive charge in ball A and are free to move in the metal.” 4. Correct answer states “This results in a larger attractive force on the electrons in ball B than the repulsive force on ball B’s protons.” |
| Crosscutting Concept: Structure and Function | |
| To code an assessment task with Structure and Function, the question asks the student to predict or explain a function or property based on a structure, or to describe what structure could lead to a given function or property. | Question asks student to explain the property that a charged object can attract an uncharged object based on the movement and location of electrons. |
| Core Idea: Interactions are Mediated by Fields | |
| Fields are generated by charges/masses. Fields affect charges/masses. In circuits, fields induce currents. | Question asks about the interaction between a charged and neutral conductor. |

P13

**Physics Example 13:** Consider the following statement made by a student in an introductory physics course about Atwood machines:

*“All strings can do is transmit forces from one object to another. Therefore, the string in an Atwood machine just simply transmits the weight of one block to the other block.”*

1. Do you agree with this student?
   1. Yes
   2. No *
2. Which of the following is the best reasoning for your answer above?
   1. If the two masses are the same, then neither block is accelerating so the force on the block from the string must equal the weight of the block.
   2. The tension in the string is always equal to the weight of the lighter block, since it requires a smaller force to undergo the same acceleration as the heavy block.
   3. The tension in the string is always equal to the weight of the heavier block, since you need the larger weight to counteract the weight of the light block and make it accelerate.
   4. The tension in the string is the same everywhere, and there is no reason for the string to transmit one block’s weight instead of the other. *

| CRITERIA | RATIONALE |
| --- | --- |
| Scientific Practice: Evaluating Information | |
| 1. Question gives an excerpt from a conversation, article, student solution, or video (or similar form of communication) that makes one or more assertions. 2. Question gives a conclusion about the validity of the assertion(s) or asks student to select a conclusion about the validity of the assertion(s) or reconciliation. 3. Question asks student to select reasoning to support their conclusion(s) about the validity of the assertion(s) or reconciliation with data, observations, or scientific principles. | 1. Question gives an excerpt of a statement “made by a student” 2. Question asks student “Do you agree with this student?” 3. Question asks student to select “the best reasoning for your answer above”. |
| Crosscutting Concept: Not Applicable | |
| Core Idea: Interactions Can Cause Changes in Motion | |
| Changes in an object’s motion are the result of interactions between it and one or more other objects. | Question involves the motion of two objects on an Atwood machine and the forces involved in the string. |
