# Supplementary material for: Characterizing College Science Assessments: The Three-Dimensional Learning Assessment Protocol
Source: PLoS One. 2016 Sep 8;11(9):e0162333. doi: 10.1371/journal.pone.0162333 (PMC5015839; doi:10.1371/journal.pone.0162333)
Supplement: S2 Supporting Information — (DOCX) [file pone.0162333.s004.docx]

**Characterizing College Science Assessments: The Three-Dimensional Learning Assessment Protocol**

James T. Laverty, Sonia M. Underwood, Rebecca L. Matz, Lynmarie A. Posey, Justin H. Carmel, Marcos D. Caballero, Cori L. Fata-Hartley, Diane Ebert-May, Sarah E. Jardeleza, Melanie M. Cooper

**Supporting Information: Skills**

We do not suggest that every assessment task should consist of all three dimensions. We recognize that there are particular skills that students must be able to do in order to answer higher-dimensional tasks which don’t necessarily meet the criteria for engaging with a scientific practice, crosscutting concept, or addressing a core idea. Because of this, tasks assessing these skills are often zero- or one-dimensional. Here, we provide examples of skills that faculty might want to assess in an introductory course that do not (on their own) meet the criteria for a scientific practice, crosscutting concept, or core idea.

- Biology
  - Drawing Punnett squares
  - Drawing molecular structures
  - Calculating heritability
  - Calculating genotype and allele frequencies/probabilities
  - Transcribing DNA to RNA
  - Translating RNA to protein
- Chemistry
  - Using scientific notation
  - Unit conversions
  - Naming/identification of elements and compounds
  - Writing electron configurations
  - Identifying core and valence electrons
  - Drawing Lewis structures
  - Calculating formal charge
  - Calculating bond order(s), average bond order
  - Determining electron pair geometry, molecular shape, bond polarity and polarity (VSEPR, vector addition)
  - Balancing chemical equations (including redox equations and nuclear reactions)
  - Stoichiometric calculations (including going between moles/number of atoms or molecules/mass/volume (for solutions and gases)and using the balanced chemical equation in calculations)
  - Determining of reaction enthalpies, entropies, and free energies, applications of Hess’ law
  - Calculating concentrations and dilution
  - Any calculation where student solves for an unknown using a standard equation (gas law, thermodynamic equations, Nernst, etc)
  - Determining oxidation numbers
  - Determining equilibrium conditions or equilibrium constants from given data
  - Determining rate laws from kinetic data
  - Translating between equations and graphs
  - Translating among multiple representations
- Physics
  - Mathematics
    - Algebra
    - Vector manipulation
    - Dot products
    - Cross products
    - Derivatives
    - Integrals
  - Identification of forces
  - Drawing free body diagrams
  - Applying Newton’s Second Law to a free body diagram
  - Drawing circuit diagrams
  - Drawing ray diagrams
  - Extracting information from graphs
  - Using “right-hand rules”
